# Mutations involving the *SRY*-related gene *SOX8* are associated with a spectrum of human reproductive anomalies

**DOI:** 10.1093/hmg/ddy037

**Published:** 2018-01-24

**Authors:** Marie-France Portnoi, Marie-Charlotte Dumargne, Sandra Rojo, Selma F Witchel, Andrew J Duncan, Caroline Eozenou, Joelle Bignon-Topalovic, Svetlana A Yatsenko, Aleksandar Rajkovic, Miguel Reyes-Mugica, Kristian Almstrup, Leila Fusee, Yogesh Srivastava, Sandra Chantot-Bastaraud, Capucine Hyon, Christine Louis-Sylvestre, Pierre Validire, Caroline de Malleray Pichard, Celia Ravel, Sophie Christin-Maitre, Raja Brauner, Raffaella Rossetti, Luca Persani, Eduardo H Charreau, Liliana Dain, Violeta A Chiauzzi, Inas Mazen, Hassan Rouba, Caroline Schluth-Bolard, Stuart MacGowan, W H Irwin McLean, Etienne Patin, Ewa Rajpert-De Meyts, Ralf Jauch, John C Achermann, Jean-Pierre Siffroi, Ken McElreavey, Anu Bashamboo

**Affiliations:** 1 APHP Département de Génétique Médicale, Hôpital Armand Trousseau, Paris 75012, France; 2 UPMC, University Paris 06, INSERM UMR_S933, Hôpital Armand Trousseau, Paris 75012, France; 3 Human Developmental Genetics, CNRS UMR3738, Institut Pasteur, Paris 75724, France; 4 Division of Pediatric Endocrinology, Children’s Hospital of Pittsburgh of UPMC, Pittsburgh, PA 15224, USA; 5 Genetics and Genomic Medicine, UCL Great Ormond Street Institute of Child Health, London WC1N 1EH, UK; 6 Department of Obstetrics, Gynecology and Reproductive Sciences, Magee-Women’s Research Institute; 7 Department of Human Genetics, University of Pittsburgh School of Medicine, Pittsburgh, PA 15213, USA; 8 Department of Pathology, University of Pittsburgh School of Medicine, Pittsburgh, PA 15213, USA; 9 University Department of Growth and Reproduction, Rigshospitalet, DK-2100 Copenhagen, Denmark; 10 Genome Regulation Laboratory, Drug Discovery Pipeline, South China Institute for Stem Cell Biology and Regenerative Medicine, Guangzhou Institutes of Biomedicine and Health, Chinese Academy of Sciences, Guangzhou 510530, China; 11 Key Laboratory of Regenerative Biology, South China Institute for Stem Cell Biology and Regenerative Medicine, Guangzhou Institutes of Biomedicine and Health, Chinese Academy of Sciences, Guangzhou 510530, China; 12 Guangdong Provincial Key Laboratory of Stem Cell and Regenerative Medicine, South China Institute for Stem Cell Biology and Regenerative Medicine, Guangzhou Institutes of Biomedicine and Health, Chinese Academy of Sciences, Guangzhou 510530, China; 13 Département Mère-Enfant, Institut Mutualiste Montsouris, Paris 75014, France; 14 Département d’Anatomie Pathologique, Institut Mutualiste Montsouris, Paris 75014, France; 15 Service d’Endocrinologie, Hôpital Cochin, Paris, France; 16 Biology of Reproduction, CHU Rennes, Rennes 35033, France; 17 Service d'Endocrinologie, Diabétologie et Endocrinologie de la Reproduction, Hôpital Saint-Antoine, Paris 75012, France; 18 Université Paris Descartes and Pediatric Endocrinology Unit, Fondation Ophtalmologique Adolphe de Rothschild, Paris 75019, France; 19 Department of Clinical Sciences & Community Health, University of Milan, Milan 20122, Italy; 20 Laboratory of Endocrine & Metabolic Research and Division of Endocrine and Metabolic Diseases, IRCCS Istituto Auxologico Italiano, Milan 20149, Italy; 21 Centro Nacional de Genética Médica, Administración Nacional de Laboratorios e Institutos de Salud (ANLIS) Dr. Carlos G. Malbrán, Buenos Aires C1428ADN, Argentina; 22 Department of Physiology, Instituto de Biología y Medicina Experimental, Consejo Nacional de Investigaciones Científicas y Técnicas (IBYME-CONICET), Buenos Aires C1428ADN, Argentina; 23 Department of Clinical Genetics, National Research Centre, Cairo 12622, Egypt; 24 Human Genetics Unit, Institut Pasteur of Morocco, Casablanca 20250, Morocco; 25 Genetic Services, Hopital Femme Mere Enfant, Bron 69677, France; 26 Centre for Dermatology and Genetic Medicine, School of Life Sciences, University of Dundee, Dundee DD1 5EH, UK; 27 Human Evolutionary Genetics, Institut Pasteur, Paris 75724, France

## Abstract

SOX8 is an HMG-box transcription factor closely related to SRY and SOX9. Deletion of the gene encoding Sox8 in mice causes reproductive dysfunction but the role of SOX8 in humans is unknown. Here, we show that SOX8 is expressed in the somatic cells of the early developing gonad in the human and influences human sex determination. We identified two individuals with 46, XY disorders/differences in sex development (DSD) and chromosomal rearrangements encompassing the *SOX8* locus and a third individual with 46, XY DSD and a missense mutation in the HMG-box of SOX8. *In vitro* functional assays indicate that this mutation alters the biological activity of the protein. As an emerging body of evidence suggests that DSDs and infertility can have common etiologies, we also analysed *SOX8* in a cohort of infertile men (*n =* 274) and two independent cohorts of women with primary ovarian insufficiency (POI; *n =* 153 and *n =* 104). *SOX8* mutations were found at increased frequency in oligozoospermic men (3.5%; *P *<* *0.05) and POI (5.06%; *P *=* *4.5 × 10^−5^) as compared with fertile/normospermic control populations (0.74%). The mutant proteins identified altered SOX8 biological activity as compared with the wild-type protein. These data demonstrate that SOX8 plays an important role in human reproduction and *SOX8* mutations contribute to a spectrum of phenotypes including 46, XY DSD, male infertility and 46, XX POI.

## Introduction

Human sex determination is a tightly controlled and highly complex process, where the bipotential gonad anlage develops as one of two mutually antagonistic fates – the ovary or a testis. *SRY* is well established as the primary testis-determining gene on the Y chromosome. In the XY gonad, *SRY* acts during embryonal development to upregulate the downstream effector *SOX9* beyond a critical threshold, which promotes testis development and in turn represses ‘ovarian pathways’ in the male gonad through multiple mechanisms (1,2). In mice, up-regulation of *Sox9* in males is by the synergistic action of Sry with Nr5a1 [also known as Steroidogenic factor-1 (Sf1)], through binding to multiple elements within a testis-specific *Sox9* enhancer (*Tesco*) (1). Sox9 expression is required to establish Sertoli cell identity in the developing testis (2). Once formed, foetal Sertoli cells produce anti-Müllerian hormone (AMH) and coordinate the cellular and morphogenetic events leading to primary sex determination (2).

Although the *SOX* genes *SRY* and *SOX9* play essential roles in driving early mammalian testis-determination, emerging evidence suggests the contribution of another *Sox* gene, *Sox8*, in murine testis-determination as well as in the maintenance of gonadal function. *Sox8* shows an overlapping expression pattern with *Sox9* in foetal and adult mouse gonad and functional redundancy between *Sox8* and *Sox9* occurs during testis development (3–6). *Sox8* XY null mutants show normal testis development but develop post-natal progressive spermatogenic failure (7).

Development and maintenance of the mammalian gonad is regulated by a double repressive system, where an equilibrium of mutually antagonistic pathways must be attained for normal development of either the testis or ovaries (2–6). In humans, changes in this delicate balance can lead to Disorders of (or Differences in) Sex Development (DSD), which are defined as congenital conditions with discordant development of chromosomal, gonadal or anatomical sex (8). The incidence of DSD has been estimated at 1: 2, 500 to 1: 5000 births (9–10). Excluding inborn errors in steroidogenesis, a molecular diagnosis will be reached in only 20% of all individuals with 46, XY DSD (9). Around 40% of the cases with 46, XY complete gonadal dysgenesis (CGD) can be explained by mutations involving three genes *SRY*, *NR5A1* and *MAP3K1* (4,5). Pathogenic variants in other sex-determining genes such as *CBX2, GATA4* or *FOG2/ZFPM2* are found in a very small percentage of cases (9,10). Hence, the aetiology in majority of the individuals with DSD remain poorly understood.

An emerging body of evidence suggests that DSD and infertility can have common aetiologies, For example, mutations involving NR5A1 (SF-1), a key player in many aspects of reproductive function including sex determination, are associated not only with a spectrum of DSD such as 46, XY CGD, 46, XY undervirilised males with testes, or 46, XX (ovo)testicular DSD but also more prevalent forms of human infertility, i.e. 46, XY men with spermatogenic failure and 46, XX women with primary ovarian insufficiency (POI) (11–14). Human infertility is a major human health issue; one in seven couples worldwide have problems conceiving and both men and women are affected equally (15,16). POI is characterised by primary or secondary amenorrhea, high gonadotropin levels (FSH above 40iU/l on two occasions at least a month apart) and estrogen deficiency in women under the age of 40 years (17). Male infertility includes azoospermia characterized by complete absence of spermatozoa in the ejaculate, whereas oligozoospermia is defined as sperm concentrations below the World Health Organization reference level of 15 × 10^6^ sperm/ml (18). The underlying basis for either male or female infertility is complex, including both physiological and environmental factors as well as gene mutations. Although mouse models have revealed hundreds of genes that are associated with fertility, only a few single-gene defects that cause male and/or female infertility have been identified in humans (12,13,19–24). Here, for the first time we demonstrate that mutations involving the human *SOX8* gene are associated with a range of phenotypes including 46, XY DSD as well as male infertility and ovarian insufficiency in females.

## Results

### Rearrangements involving the *SOX8* locus and 46, XY DSD

**Figure 1.**
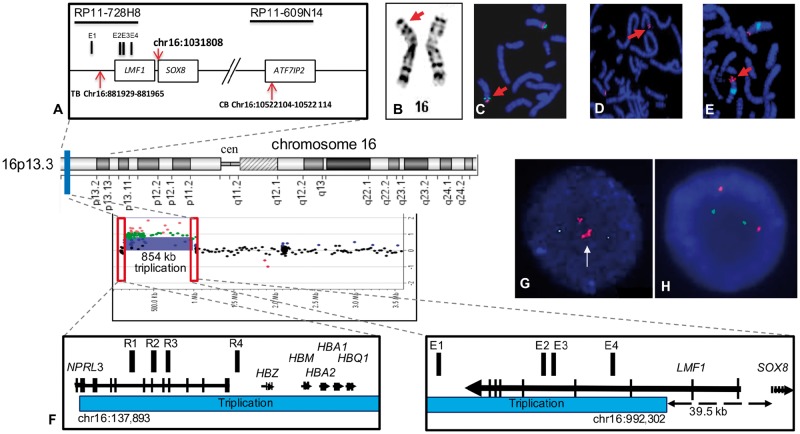
Chromosomal rearrangements at the *SOX8* locus associated with 46, XY DSD. (**A**) Schematic representation of the 16p inversion in patient 1 indicating the genes and clones of the region and the position of the telomeric breakpoint (TB) and centromeric breakpoints (CB). The position of the transcription initiation site of the *SOX8* gene is indicated (chr16: 1 031 808), the *Sox8* enhancer elements (E1–E4) and the position of the BACs used for FISH analysis. Patient 1 with (**B**) GTG banding showing the rearranged chromosome 16 (arrow). (**C**) FISH on metaphases with BAC RP11–161M6 (green; chr16: 991, 108–1, 141, 991- UCSC Genome Browser; GRCh37/hg19) and RP11–297M9 (red; chr16: 9, 727, 655–9, 920, 250) confirming the paracentric inversion (arrow). (**D**) The CB is located within the clone, RP11–609N14 on 16p13.13). (**E**) The TB mapped within RP11–728H8, on 16p13.3 (arrows). Patient 2 (**F**) Chromosome 16p rearrangement. The aCGH profile indicated an 854 kb triplication in the 16p13.3 region. Affected region is indicated by a blue bar. Red rectangles outline the triplication breakpoints. A magnified view of the triplication breakpoints shows, on the left, a CB located in the intron 1 of the *NPRL3* gene. The 16p13.3 triplication starts at chr16: 137893 position and spans proximally including the α-globin genes (*HBZ*, *HBM*, *HBA1*, *HBA2*, and *HBQ1*) and their upstream cis-regulatory elements R1-R4 (red bars). On the right, the TB of the triplication (chr16: 992, 302) is located ∼39.5 kb upstream of the *SOX8* gene. The *SOX8*-specific enhancers are mapped within the triplicated segment. Genes are indicated by black arrows with vertical lines specifying exons. Arrows correspond to a direction of gene transcription. (**G**) Interphase FISH analysis using RP11–598 I20 (16p13.3, labeled red) and a control RP11–121O8 (16p13.1, labeled green) probe showing a triplication (white arrow) in the patient 20. (**H**) Representative FISH image using the same probes shows a normal hybridization pattern observed in both parents.

**Figure 2.**
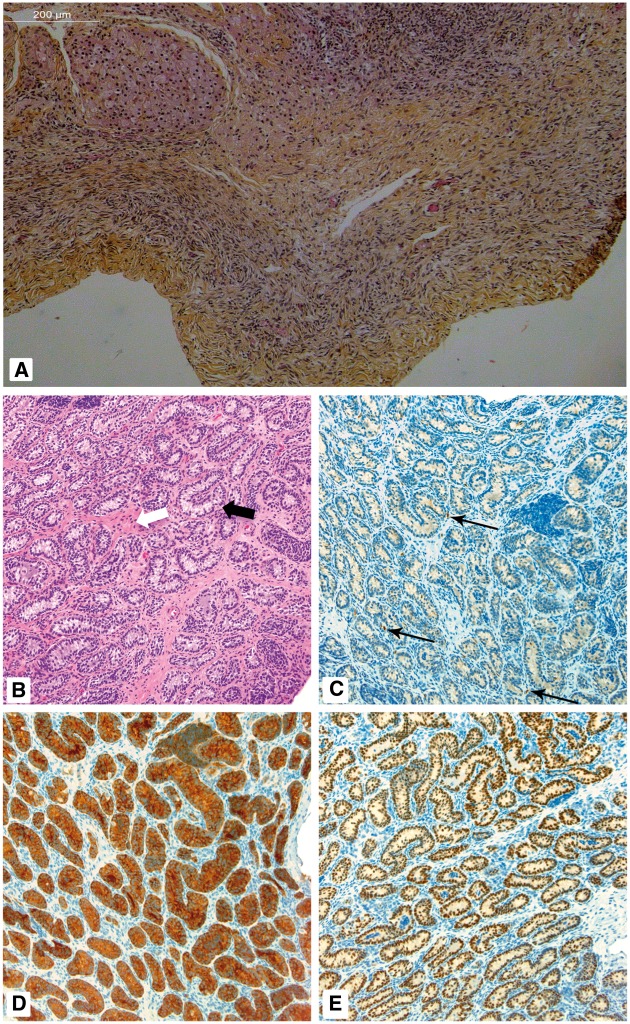
Gonad histology of Patients 1 and 2. Patient 1, (**A**) Ovarian-like stromal cells with no evidence of testicular tissue consistent with 46, XY complete gonadal dysgenesis. Patient 2, (**B**) Seminiferous tubules (cords) are mildly contorted in places (black arrow) and some are misshapen. The interstitium space is expanded and mildly fibrotic (white arrow; H&E; 200X). (**C**) Immunohistochemistry for calretinin shows only focal Leydig cells (arrows; calretinin stain, blue; 200X). (**D**) Inhibin immunohistochemistry highlights Sertoli cells, making the contorted tubules more evident (inhibin; 200X). (**E**) Immunohistochemistry for SOX9 staining the nuclei of Sertoli cells within tortuous tubules (SOX9; 200X).

Patient 1 is a phenotypic female who presented at the age of 27 years with primary amenorrhea (Patient 1, Table 1). Chromosome analysis showed a 46, XY karyotype with a paracentric inversion [46, XY, inv(16)(p13.3p13.1); Fig. 1A and B], which was confirmed by FISH analysis (Fig. 1C). Her parents and unaffected sister were not available for analysis. Hence, it is unknown if this rearrangement is *de novo*, however, this rearrangement has not been reported in control databases. Array-CGH did not reveal any chromosomal imbalances, including the chr16 short arm, associated with the phenotype. The centromeric breakpoint was mapped within the BAC clone RP11–609N14 (chr16: 10428838–10600163) (Fig. 1D). The telomeric breakpoint was mapped within the BAC clone RP11–728H8 between chr16; 814 190–962 809 (Fig. 1E). Whole genome sequencing confirmed these results and indicated a centromeric breakpoint at chr16: 10 522 104–10 522 114 and a telomeric breakpoint at chr16: 881 929–881 965. The transcription initiation site of the *SOX8* gene is at chr16: 1 031 808. Therefore, the breakpoint is located ∼150 Kb upstream of *SOX8* (Fig. 1A). A bilateral gonadectomy revealed two small gonads, which on histological examination showed two streak-like gonads with no germ cells or differentiated tissue (Fig. 2A).

**Table 1.** Phenotypes, genetics and clinical investigations of 46, XY DSD with mutations involving the *SOX8* locus (patients 1 and 2) or gene (patient 3)

Patient	Phenotype	Clinical presentation (age)	Genetics	Hormonal profile(normal range)	Genitalia and gonads	Diagnosis
1	Female	Primary amenorrhea with female external genitalia	46, XY, inv(16) (p13.3p13.1)	FSH: 59.5 iU/l (2.0–15.0)	Bilateral streak gonads with fallopian tubes. Stromal-like tissue with no evidence for testicular tissue or follicles.	46, XY CGD
				LH: 23.6 iU/l (2.1–14.9)	Small immature uterus, 43 × 22×30 mm (27 years).	
				T: 0.7 ng/ml (2.70–9.00)		
2	Female at birth then changed to male assignment	Ambiguous genitalia (birth), mild anaemia, skeletal anomalies, mild developmental delay	46, XY	FSH: 2.31 iU/l (0.16–4.1 mIU/ml)	At birth clitorophallic structure (1 × 1cm) with urethral meatus at base. Fused labioscrotal folds with palpable gonads (R: 1.2 × 0.6 × 0.8cm, L: 1.1 × 0.4 × 0.7cm). No uterus or vagina. Testis histology showed seminiferous tubules, variable germ cells and reduced Leydig cell numbers.	46, XY severe undervirilization
			arr[h19] 16p13.3 (137, 893–992, 302)×3	LH: 17.35 iU/l (0.02–7.0 mIU/ml)		
				T: 1.38 ng/ml (0.6–4 ng/ml)		
				DHT 36 ng/dL (12–85)		
3	Female	Primary amenorrhea (16 years)	46, XY SOX8	Elevated FSH, LH: undetectable T levels	Bilateral streak gonads with absent uterus. Epididymis-like structure present.	46, XY PGD
			p.Glu156Asp			
			PolyPhen2 Score			
			0.999			

CGD: complete gonadal dysgenesis; PGD: partial gonadal dysgenesis; FSH: Follicle stimulating hormone; LH: Luteinizing hormone. All mutations are heterozygous.

**Figure 3.**
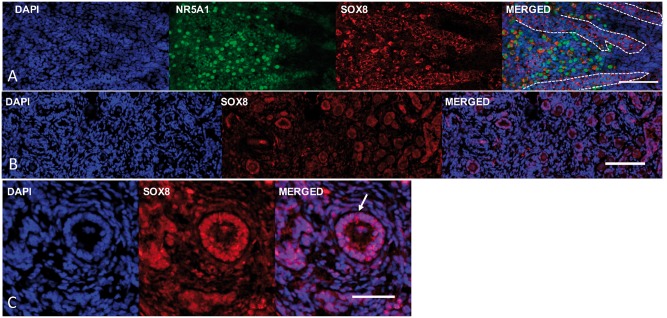
Expression of the SOX8 protein in human gonad tissues. (**A**) The SOX8 protein (red) is expressed in the human male Sertoli and Leydig cells together with NR5A1 (green) during early testis formation (image at 9 weeks post-conception). Primitive seminiferous cords are indicated by the dashed lines (**B**) The SOX8 protein expression in the granulosa cells of the late fetal ovary (image at 40 weeks of gestation). (**C**) Immunohistochemistry showing SOX8 expression in granulosa cells (arrow) lining the follicles of the adult ovary (19 years old; scale bars are 50 μm).

Patient 2 is a 46, XY girl with mild post-natal anaemia, sleepiness, poor feeding and tachycardia (Patient 2, Table 1 and Supplementary Material). Physical examination showed a prominent forehead and ophthalmological evaluation revealed small bilateral chorioretinal colobomas. At birth the genitalia consisted of a midline clitorophallic structure with bilateral labioscrotal folds, which were fused at the midline. On pelvic ultrasound, there was no uterus or vagina noted and gonads and epididymides were identified in the labia bilaterally. Pituitary hormone levels were within normal limits. The child was assigned male and at 10 months of age underwent the first stage of surgery for hypospadias, chordee repair and posterior urethral mobilization, and scrotoplasty. At 2 years 10 months the patient underwent bilateral orchidopexy, bilateral inguinal hernia repair, and bilateral gonadal biopsy. Gonadal histology showed seminiferous cords, variable germ cells and reduced Leydig cell numbers by calretinin staining (Fig. 2B–E). aCGH detected a two copy DNA gain in the 16p13.3 region [arr(h19) 16p13.3(137 893–992 302]×4, log_2_=+1), spanning at least 854 kb (Fig. 1F), resulting in tetrasomy of that DNA segment. This region comprises at least 27 genes, including the α-globin gene cluster (Supplementary Material, Table S1). FISH analyses using RP11–598I20 BAC probe on cultured peripheral blood samples from the patient and both parents indicated that the tetrasomy was a *de novo* event (Fig. 1G and H). The centromeric breakpoint of this triplication was located within the intron 2 of the *LMF1* (lipase maturation factor 1) gene, ∼39.5 kb upstream of the *SOX8* gene. Homozygous, loss-of-function *LMF1* gene mutations are responsible for hypertriglyceridemia and decreased lipase activity and hence this gene was not considered to be associated with the phenotype (25). Interestingly, *SOX8*-specific enhancer elements are included in the triplication (26). Together, these data suggest that rearrangements at the *SOX8* resulting in dysregulation of *SOX8* expression could negatively impact testis-determination and may result in 46, XY DSD including disorders of testis-determination. Consistent with this postulation, we observed that SOX8 is co-expressed with NR5A1 and SOX9 in the early stages of human testis-determination in Sertoli cells and Leydig cells as well as in Sertoli and Leydig cells in adult men (Fig. 3A, Supplementary Material, Fig. S1). Single cell RNA sequencing on XY mouse gonads during sex determination has also demonstrated that *Sox8* is co-expressed with other genes involved in sex determination including *Nr5a1*, *Dmrt1* and *Gata4* (27). 

### A missense mutation in the HMG-box domain of SOX8 is associated with a lack of testis-determination

**Figure 4.**
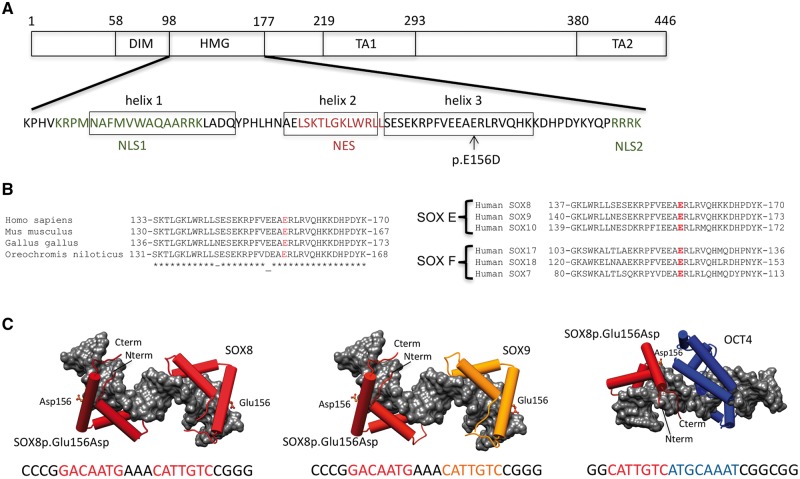
Mutation in the HMG-box of SOX8 associated with 46, XY gonadal dysgenesis. (**A**) Schematic representation showing important functional domains of the SOX8 protein. The amino acid sequence of the HMG domain including the three alpha-helices, the two nuclear localization signals (NLS1, NLS2), and the nuclear export sequence (NES) together with the position of SOX8p.Glu156Asp (p.E156D) mutation are indicated. The DNA-dependent dimerization domain (DIM), the DNA-binding HMG domain, and the transactivation domains (TA1, TA2) are shown. (**B**) Left, alignment of the C-terminal of the SOX8 HMG-box domain containing helix 3 from selected vertebrates. Right, the alignment of the distal portion of the SOX8 HMG-box domain with other human SOXE and SOXF family members. The position of the p.Glu156Asp missense mutation is highlighted. (**C**) Structural models for ternary complexes of conjectured SOX8p.Glu156Asp -SOX8, SOX8p.Glu156Asp -SOX9 and SOX8p.Glu156Asp -OCT4 dimers on composite DNA elements. SOX8 is shown in dark red, SOX9 in orange and OCT4 in blue. The HMG domains of SOX E proteins and the POU domain of OCT4 are shown as cartoon with cylindrical alpha-helices and the DNA as gray van-der-Waals surface. The SOX8p.Glu156Asp site is drawn as ball-and-sticks. The DNA sequences used to generate the models with colour-coded binding elements are depicted underneath the models. The SOXE HMG box is N-terminally extended by a 40 amino acid dimerization (DIM) domain of unknown structure that mediates highly cooperative DNA-dependent dimerization presumably by interactions with the HMG box.

Based on the above observations, we extended the *SOX8* mutation screen to a large cohort of 46, XY DSD individuals (Table 2). Three heterozygous missense *SOX8* variants were identified in a screen of 204 cases of unexplained 46, XY DSD. Two of these changes were observed in the control cohort and their contribution to the phenotype is considered unlikely. The third mutation, a heterozygous c.468G > C p.Glu156Asp amino acid substitution was identified in a 46, XY phenotypic female who presented at 16 years of age with primary amenorrhea (Patient 3 Table 1, Fig. 4A). The Glu156 residue is located in the HMG-box and is evolutionarily conserved not only within the SOX8 protein but also in other members of groups E and F SOX protein family (Fig. 4B). The mode of inheritance of this mutation is unknown as the parents were unavailable for study. This mutation was not observed in our control cohort, but it is present as a rare variant in the ExAC database (2: 121, 228 alleles, rs764098477). The SOX8 p.Glu156Asp mutation is not expected to disrupt DNA binding *per se* because this residue does not map to the DNA interaction interface (Fig. 4C). Furthermore, exchanging the equivalent residues in Sox2 (Sox2Lys to Glu) and Sox17 (Sox17Glu to Lys) are reported not to show any effect on DNA binding by the SOX HMG box in the absence of partner proteins (28). However, this mutation could affect how SOX8 dimerizes with its partner factors on DNA, since changing the same residue in Sox2 and Sox17 affected the ability of these proteins to interact with OCT4 [Fig. 4C (28)].

**Table 2.** *SOX8* mutations in 46, XY DSD, male infertility, primary ovarian insufficiency and in the control cohort of ancestry-matched fertile/normospermic individuals

46, XY DSD (*n =* 204)	Azoospermia (*n =* 131)	Oligozoospermia (*n =* 143)	POI (*n =* 153)	POI replication study (*n =* 104)	Fertile and/or normospermic controls (*n =* 813; European ancestry 503; North African/Arab ancestry 310)
p.Glu156Asp	p.Lys241Thr	p.Gly359Arg (x2)	p.Arg8_Ser9deldeletion of 2 AA	p.Pro11Leu	p.Ala240Thr^d^
p.Pro336Leu	p.Asp382Asn	p.Ala373Ser	p.Ala32Val	p.Pro196Leu	p.Thr331Met^e^
p.Thr340Ser		p.Gly378Ser	p.Pro242Leu (x2, sisters)	p.Pro242Leu	p.Pro336Leu^f^
		p.Asp382Asn	p.Ser267Leu	p.Ala416Thr	p.Thr340Ser^f^
			p.Val282Ile		p.Ala373Thr^d^
			p.Asp382Asn (x3)		p.Ala428Thr^f^
			p.Ala416Thr		
1.47%	**1.53%**	**3.5%**^a^	**5.9%**^b^	**3.85%**^c^	**0.74%**

a *P = *0.0151, ^b^*P* = 0.000107, ^c^*P* = 0.0191 compared with controls (Fishers *t*-test, two-tailed), POI: Primary ovarian insufficiency; DSD: Disorder of Sex Development; AA: Amino acids. All mutations are heterozygous. Fertile fathers from, ^d^1000 genomes project (European ancestry), ^e^Danish Genome Project and ^f^in-house controls.

**Figure 5.**
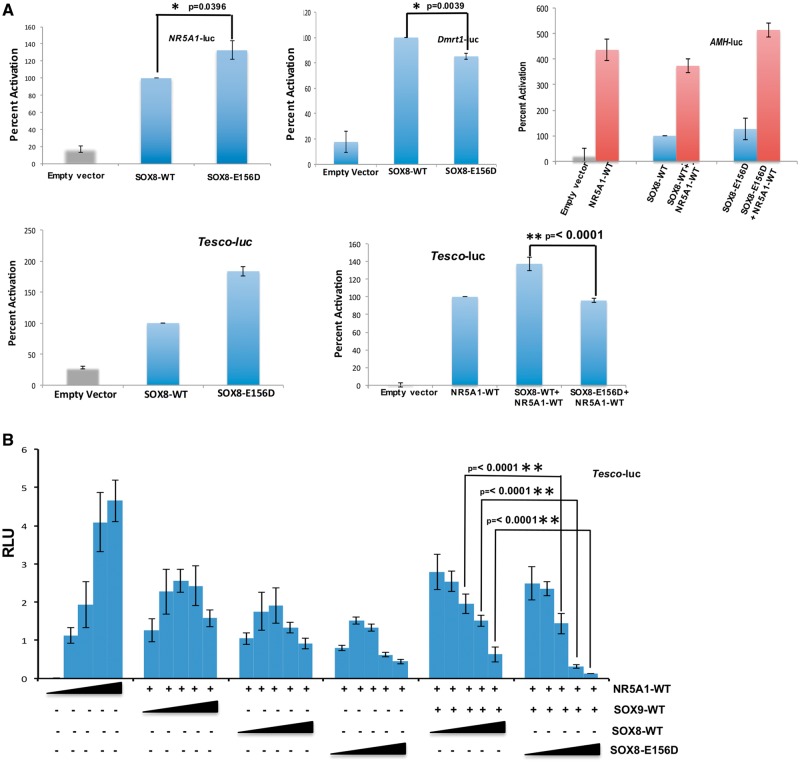
SOX8p.Glu156Asp shows altered biological activity. (**A**) The transcriptional activities of WT-SOX8 and SOX8p.Glu156Asp were studied using the human *AMH* and *NR5A1* promoters, mouse *Dmrt1* promoter and the *Sox9 Tesco* enhancer as reporters, following transfection in HEK293-T cells. The data shown represent the mean ± SEM of minimum three independent experiments, each of which was performed at least in quadruplicate. The reporter constructs were transfected into HEK293-T cells with either the WT-SOX8 or SOX8p.Glu156Asp expression vector. The results are expressed as relative percentage of WT-SOX8 activity (100%). The SOX8p.Glu156Asp can activate the gonadal promoters in a similar fashion to WT-SOX8. However, although SOX8p.Glu156Asp can synergistically activate the *AMH* promoter with NR5A1, it fails to synergise with *NR5A1* to activate the *Sox9 Tesco* enhancer. (**B**) HEK 293-T cells were co-transfected with *Tesco* reporter construct (10ng), WT-NR5A1 (1ng) and WT-SOX9 (1ng) and increasing amount of WT-SOX8 or SOX8p.Glu156Asp (0, 1, 2, 5, 10 ng). Results are expressed as Relative Luminescence Units (RLU). The SOX8p.Glu156Asp mutant exerts a repressive effect by preventing synergistic activation of the *Sox9 Tesco* enhancer by NR5A1 and SOX9 even at three times lower concentrations.

**Figure 6.**
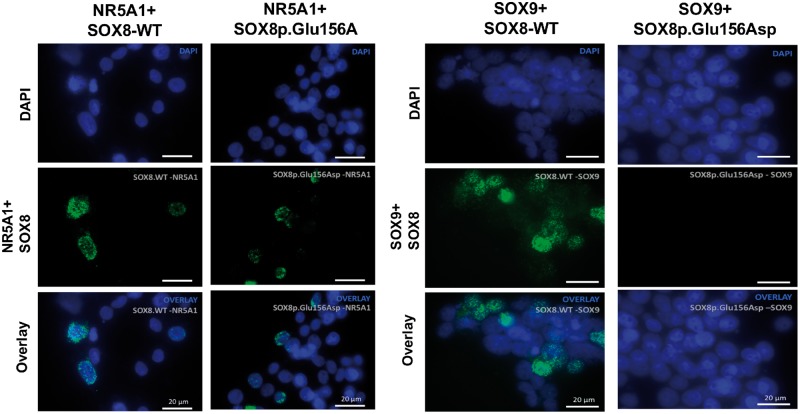
Protein–protein interaction assayed using Proximity ligation assay. Plasmids encoding SOX8-WT or SOX8p.Glu156Asp were transiently expressed with WT-NR5A1 or SOX9 for 48 h in HEK293T cells. Protein–protein interaction of SOX8-WT and SOX8p.Glu156Asp with NR5A1 and SOX9 were analysed using the Duolink proximity ligation assay. Nuclei are stained with DAPI (blue) and Duolink signal representing interaction between the proteins is shown in green. Each green dot represents a single dimerization event. Both the SOX8-WT and SOX8p.Glu156Asp proteins can physically interact with NR5A1. Whereas, although SOX8-WT protein can interact with SOX9, the SOX8p.Glu156Asp cannot.

A series of *in vitro* analyses were performed to assess the effect of this mutant on the biological activity of the SOX8 protein. Transient transfection assays were performed since a number of *SOXE* group-responsive genomic elements have been identified that, when placed upstream of a reporter gene, can be used to quantitate transcriptional regulatory function of protein variants (29). The function of *SOX* genes involves a complex interaction with many other transcriptional co-regulators, including other SOX proteins (29) and therefore we considered that the consequence of the *SOX8* mutation may be promoter or cell context dependent. Our data show that the mutant SOX8 protein exhibits a context-specific loss-of-function activity. The WT-and mutant SOX8 proteins can activate a series of gonadal promoters (Fig. 5A). However, the SOX8p.Glu156Asp specifically fails to synergize with NR5A1 to transactivate the *Sox9 Tesco* enhancer element (Fig. 5A), yet both WT-SOX8 and SOX8p.Glu156Asp proteins physically interact with the NR5A1 protein (Fig. 6, Supplementary Material, Fig. S2). The mutation also impacted on the functional interaction between SOX8 and SOX9. Both SOXE proteins have the ability to heterodimerize and combinatorially regulate their target gene expression (30,31). We show that the WT-SOX8 protein binds to SOX9, however the SOX8p.Glu156Asp protein cannot (Fig. 6, Supplementary Material, Fig. S2). The SOX8p.Glu156Asp mutant also exerts a repressive effect by negatively affecting the synergistic activation of the *Tesco* enhancer by NR5A1 and SOX9 (Fig. 5B). Finally, the SOX8p.Glu156Asp protein also has a dominant negative effect on the WT-SOX8 protein since it reduced, in a dose-dependent manner, the synergistic activation of the Tesco reporter by WT-SOX8 and NR5A1 (Supplementary Material, Fig. S3). All these results are consistent with the hypothesis that the mutant SOX8 protein has an altered biological activity impairs testis-determination. 

### *SOX8* mutations associated with human infertility

To investigate whether mutations in *SOX8*, like *NR5A1*, could also contribute to a wider spectrum of male and female infertility, we sequenced the coding region of *SOX8* in 427 men and women with infertility. The incidence of mutations in the control cohort of fertile and/or normospermic men was 0.74%. In contrast, rare or novel *SOX8* variants were observed in 1.47% of azoospermic men, 3.5% of oligozoospermic men (*P *=* *0.0151, Fishers *t*-test, two-tailed) and 9/153 women with POI in an initial cohort (5.9%; *P *=* *0.000 107) and in 4/104 (3.85%; *P *=* *0.0191) of women with POI in a replication cohort (Tables 2 and 3). Combining the data from both cohorts, we found a significant association of mutations in *SOX8* with POI (5.06%; *P *=* *4.5×10^−5^).

**Table 3.** Mutations in the *SOX8* gene associated with human male or female infertility. Heterozygous missense mutations were identified in the *AIRE* and *NR5A1* genes in cases 18 and 20, respectively. In such cases the *SOX8* mutation may contribute to the severity of the phenotype

Patient	Mutation	Variant identifier	Global minor allelic frequency	PolyPhen2 Score	Ancestry	Phenotype and comments
4	p.Lys241Thr	rs553836905	0.0002	0.762	European	Azoospermia
5	p.Gly359Arg	rs199719055	0.000014	0.961	Eastern European	Oligozoospermia 3.4 × 10^6^/ml
6	p.Gly359Arg	rs199719055	0.000014	0.961	French	Oligozoospermia 2 × 10^6^/ml
7	p.Ala373Ser	rs200828189	0.0036	0.000	French	Oligozoospermia 9 × 10^6^/ml
8	p.Gly378Ser	rs576173763	0.0064	0.004	Senegalese	Severe oligozoospermia 0.005 × 10^6^/ml
9	p.Asp382Asn	rs143203270	0.0057	0.986	Tunisian	Oligozoospermia. 6 × 10^6^/ml
10	p.Asp382Asn	rs143203270	0.0057	0.986	Algerian	Azoospermia
11	p.Arg8_Ser9del^a^	—	—	—	Italian	POI. Menarche at 13 years; one pregnancy; secondary amenorrhea and POI at 37 years. Mother had menopause at 35 years
12	p.Pro11Leu	rs200231150	0.010	0.636	European	POI. Primary amenorrhea
13	p.Ala32Val*	—	0.000009	0.919	Italian	POI. Menarche at 14 years; secondary amenorrhea and POI at 21 years
14	p.Pro196Leu	—	0.0000275	0.001	European	POI. Primary amenorrhea. Atrophic ovaries
15	p.Pro242Leu	rs567451602	0.0057	0.001	Paraguay	POI. Primary amenorrhea
16	p.Pro242Leu	rs567451602	0.0057	0.001	Paraguay	POI. Primary amenorrhea (sister of case 12)
17	p.Pro242Leu	rs567451602	0.0057	0.001	European	POI. Primary amenorrhea. Atrophic ovaries
18	p.Ser267Leu	rs368972027	0.000017	0.942	Italian	POI. Amenorrhea at 30. Hypoparathyroidism during childhood
19	p.Val282Ile^a^	—	—	0.990	Argentinian	POI. Secondary amenorrhea
20	p.Asp382Asn	rs143203270	0.0057	0.986	North African	POI. Menarche at 12 years; secondary amenorrhea at 14 years.
21	p.Asp382Asn	rs143203270	0.0057	0.986	European	POI. Primary amenorrhea at 16 year. Absent pubertal development, ovaries not seen by ultrasound
22	p.Asp382Asn	rs143203270	0.0057	0.986	French	POI. Menarche at 11 years; secondary amenorrhea at 14 years
23	p.Ala416Thr	rs201602067	0.0004	0.005	Italian	POI. Menarche at 9 years; secondary amenorrhea and POI at 38 years
24	p.Ala416Thr	rs201602067	0.0004	0.005	European	POI. Primary amenorrhea

a Novel variant.

POI, primary ovarian insufficiency. All mutations are heterozygous.

The majority of mutations associated with infertility flank the HMG-box and fall within one of the two transactivation domains. Although many of these missense mutations are predicted by computational methods to have a deleterious effect on protein function, the interpretation of the significance of mutations in human disease remains very challenging. We therefore sought to test the consequence of the *SOX8* mutations associated with infertility in a series of functional assays. As expected, the transient transfection assays using the *AMH*, *Dmrt1* and *NR5A1* promoters as reporters in HEK 293-T and mES cells indicated biological differences between mutated wild-type proteins that, in some cases, were promoter specific, e.g. the p.Asp382Asn mutation shows a specific loss-of-function with the *AMH* promoter (Supplementary Material, Fig. S4). Furthermore, some mutant proteins show changes in cellular localization (Supplementary Material, Fig. S5).

Although, a contribution of Sox8 to male infertility in the mouse has been established for some time, the finding of an association between human POI and SOX8 variants is a novel finding since *Sox8*^−^^/^^−^ female mice are fertile (7). We therefore sought to examine the profile of SOX8 expression in the human ovary to see if this is consistent with a role for mutations in the protein contributing to female infertility. We observed that the SOX8 protein is expressed in the 40-week foetal ovary and in the adult ovary (Fig. 3B and Supplementary Material, Fig. S1). The SOX8 protein is highly expressed in granulosa cells at both stages.

## Discussion

Our data provide the first evidence that SOX8 plays a role in human sex determination and in the function of the adult gonad. Mouse models also suggest that Sox8 plays a key role in early testis-determination as well as maintaining male fertility. We identified three patients with 46, XY DSD who carried rearrangements/mutations involving the *SOX8* gene.

One patient had a paracentric inversion with a breakpoint ∼150 Kb upstream of the *SOX8* gene. The only other gene within the region is *LMF1*. Since, homozygous, loss-of-function mutations in the *LMF1* gene are associated with hypertriglyceridemia and decreased lipase activity, this gene was not considered to be responsible for the phenotype (25).

A second patient carried four copies of an 854 Kb region immediately 5ʹ to the *SOX8* gene including *SOX8* enhancer elements as well as the α-globin gene cluster (26). Duplication of α-globin genes is a rare cause of anaemia that may lead to imbalances of α- and β-chains in haemoglobin tetramer, especially in β-thalassemia carriers (32,33). Alternatively, the 16p13.3 chromosomal rearrangement may result in disruption of the long-range regulation leading to a partial silencing of gene expression consistent with prenatal and postnatal anaemia observed in patient 2 (34). The other somatic anomalies seen in this patient may be a result of gene dosage of one or more of the other genes located in the duplicated region (Supplementary Material, Table S1). Indeed, a patient with 46, XY gonadal dysgenesis, skeletal and cardiac anomalies and developmental delay was reported to carry a similar 560 kb duplication located approximately 18 kb upstream of *SOX8* (35). These rearrangements at the *SOX8* locus may cause dysregulation of *SOX8* expression. This hypothesis is consistent with the multiple reports of 46, XY gonadal dysgenesis associated with chromosomal rearrangements even up to several Mb upstream of genes involved in sex determination including *SOX9* and *NR0B1* (36–39). Furthermore, the centromeric breakpoint in patient 2 falls between the conserved E1 and E2 *SOX8* enhancer elements, which are required for murine *Sox8* gene expression (26). Dysregulation of *SOX8* expression could negatively impact on male gonadal development and lead to various degrees of 46, XY DSD. Indeed, 46, XY individuals carrying deletions that include *SOX8* (ATR16 syndrome) occasionally present with mild anomalies such as hypospadias or cryptorchidism and Patients 1 and 2 may represent part of this broad DSD spectrum (40).

**Figure 7.**
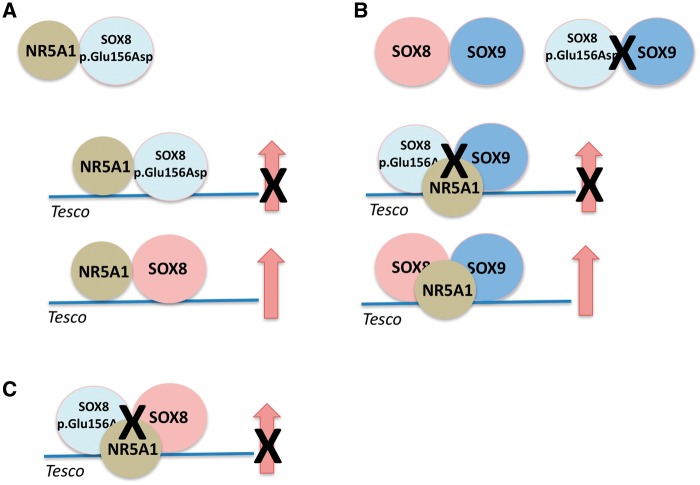
Schematic representation of the results of the in vitro assays for the SOX8p.Glu156Asp protein. (**A**) The mutant SOX8 protein has the ability to physically interact with the NR5A1 protein but in contrast to the WT-SOX8 protein does not synergize with NR5A1 to promote reporter gene activity using the *Tesco* enhancer as a target. (**B**) The mutant SOX8 protein does not physically interact with the SOX9 protein and shows a lack of synergy with SOX9/NR5A1 to promote reporter gene activity. (**C**) The SOX8 mutant protein also shows dominant negative activity by impairing promoter gene activity of the WT-SOX8 protein in synergy with NR5A1.

The third patient presenting with 46, XY gonadal dysgenesis had a missense mutation involving a highly conserved glutamic acid residue within helix 3 of the *SOX8* HMG-box. The severe gonadal phenotype in this patient, which is due to lack of appropriate testis-determination, may be due to the specific dominant negative activity of the SOX8p.Glu156Asp protein on the WT-SOX8 protein and/or the repressive effect of this mutant protein on SOX9/NR5A1 synergy at the *Sox9 Tesco* enhancer that we observed using *in vitro* assays (Fig. 7). These data, which provide evidence for a role of *SOX8* in early human testis development are supported by murine studies. The absence of both *Sox9* (pro-testis) and *Rspo1* (pro-ovary) in XX foetuses results in the development of ovotestes and hypoplastic testis (4). *SoxE* group genes, *Sox10* and *Sox8* that are normally repressed by Rspo1, are activated in the double knockout gonads, suggesting that Sox8 may serve as a driver of testis formation in the absence of Sox9 (4). Recent data suggest that Sox8 may also be involved in repressing the ovarian pathway, via the repression of Foxl2 expression during early testis formation (41). 

In this study, we have also identified mutations in *SOX8* associated with cases of male and female infertility. Although the mechanism(s) leading to infertility in either sex is unclear, all mutations associated with infertility showed differences in biological activity compared with the WT protein and all mutations flank the HMG-box. Transient transfection assays using as a target the promoters of genes important for gonad function showed a wide range of effects including promoter specific loss-of-function or alterations in the cellular localization of the mutated protein. Inferring precisely how these mutations cause infertility is difficult since the gene regulatory pathways downstream of SOX8 have not been defined in either the XY or XX gonad. In mice *Sox8* is essential for the maintenance of male fertility beyond the first wave of spermatogenesis (10). At 5 months of age *Sox8*^−/−^ mice show a progressive degeneration of the seminiferous epithelium through perturbed physical interactions between Sertoli cells and the developing germ cells (10). Here, we identified rare or novel *SOX8* mutations in 3.5% (*P *<* *0.05) of men with unexplained reduced sperm counts as compared with a frequency of 0.78% in ancestry-matched normospermic/fertile control cohorts. It is important to compare our data with a cohort of individuals who are known to be fertile and/or normospermic since infertility is a common phenotype and public databases, such as ExAC, are likely to contain a background pool of rare variants that can cause infertility and/or DSD. These cases of male infertility may represent mild forms of testicular dysgenesis or, similar to the mouse model the SOX8 protein may also be a regulator of Sertoli–germ cell adhesion independently of its role in primary testis-determination (10).

Our analyses also revealed mutations in *SOX8* in 5.06% of all women with POI from two replication studies (*P *=* *4.5×10^−5^). This suggests that mutations in human *SOX8* may have a greater impact on ovarian rather than testis function. Although *Sox8*^−/−^ female mice are fertile, *Sox8* expression has been reported in preantral follicles, preovulatory follicles, cumulus granulosa cells and at high levels in mural granulosa cells, which line the wall of the follicle and are critical for steroidogenesis and ovulation (7,42). We have shown that SOX8 protein is expressed specifically in the granulosa cell of the developing and adult ovary in the human. The observation that *Sox8* expression is higher in the mural cells that line the follicle wall than in the cumulus cells that surround the oocyte suggests that SOX8 may play a role in granulosa cell differentiation (42). We can hypothesize that in female, in the absence of SOX9, SOX8 may be an important regulator of AMH expression (required for maintenance of the germ cell pool) in the adult ovary and, therefore, mutations in *SOX8* may result in POI.

The results presented in this study provide novel insight into the genetic mechanisms of human gonadal development and function and provide further evidence that a spectrum of reproductive phenotypes from DSD to infertility can be associated with variations in a single genetic factor.

## Materials and Methods

### Detailed study methods are provided in SI methods

#### Subjects and samples

All patients with 46, XY DSD met the revised criteria of the Pediatric Endocrine Society (LWPES)/European Society for Paediatric Endocrinology (ESPE). This study was approved by the local French ethical committee (2014/18NICB – registration number IRB00003835) and consent to genetic testing was obtained from adult probands or from the parents when the patient was under 18 years. Patient ancestry was determined by self-reporting, based on responses to a personal questionnaire, which asked questions pertaining to the birthplace, languages and self-reported ethnicity of the participants, their parents and grandparents. Genes known to be involved in 46, XY DSD were screened for mutations in the XY DSD cohort and high-resolution aCGH was performed on all cases and indicated normal ploidy in all cases. An extended description of patient 2 is provided in SI methods. The infertile male population consisted of 274 men of European or North African ancestry. For each man, ejaculates were obtained by masturbation after 2–7 days of sexual abstinence. All underwent an andrological work-up, which included medical history, physical examination, hormonal evaluation (FSH, LH, and testosterone) and semen analysis. Men with known clinical (cryptorchidism, infections, varicocele) or genetic (karyotype anomalies, Y chromosome microdeletions) causes of infertility were excluded. Oligozoospermia was defined as having less than 15×10^6^ sperm/ml. Primary amenorrhea was diagnosed when pubertal development was absent despite the patient being of pubertal age (greater than 13 years) with increased basal plasma FSH concentration (>9 IU/l). Cases of secondary amenorrhea (no menstruation after six cycles) and premature ovarian failure (amenorrhea, hypoestrogenism, and elevated serum gonadotropin levels in women younger than 40 years of age) were included.

The control panel consisted of 280 unrelated normospermic 46, XY males of French ethnic origin with known fertility and no history of testicular anomalies (determined by self-reporting) from the Biobank for Research on Human Reproduction (GERMETHEQUE). An additional in-house 180 normospermic men and 130 fertile men of Arab/North African ancestry were included as controls. European ancestry-matched in-house controls of known fertility status were also included (*n =* 20). Other control populations included fathers of European ancestry from the 1000 genomes project (Iberian and Northern Europeans from Utah, *n =* 103), the Danish Genome Project (*n =* 50) and from the University of Dundee (*n =* 50).

#### Genomic analysis

Chromosome analysis, ArrayCGH, genomic, exome and Sanger sequencing are all described in SI Materials and Methods.

#### Immunohistochemistry

Human fetal testis tissue (9 weeks post-conception) was provided with approval from the Human Developmental Biology Resource (HDBR, www.hdbr.org). Term fetal (40 weeks) ovary was obtained from Abcam (#ab4412) and adult ovary tissue (19 years) was obtained from Tissue Solutions. Tissue sections (12 µm) were fixed briefly in 4% PFA in TBS, rinsed in TBS and blocked in 1% BSA in TBS-Tween (0.5% Tween) for 1 h before incubating overnight with rabbit monoclonal SOX8 antibody (Sigma HPA41640; 1: 200 dilution) and, where relevant, mouse monoclonal NR5A1 (SF1) antibody (Thermo Fisher #434200; 1: 200 dilution). Sections were washed in TBS-Tween and incubated for 1 h with the relevant secondary antibodies: Alexa555 goat anti-rabbit (Invitrogen, A21429; 1: 400) and Alexa488 goat anti-mouse (Invitrogen, A11001; 1: 400). Nuclei were counterstained with DAPI (Sigma). Slides were washed and mounted using ProLong Gold Antifade Mountant (Lifetech, #P36930). Images were collected on a Zeiss LSM 710 confocal microscope (Carl Zeiss) and analysed using Zeiss Zen 2009 and Image J.

Details of the plasmids, cell lines, cellular localization assays, structural modelling and co-immunopreciptation are provided in the SI methods.

#### Statistics

The results of the luciferase assays were compared for their statistical significance by calculating two-tailed Student’s *t*-test using GraphPad Prism software. 95% confidence interval was calculated using the mean difference between the two groups being tested. The data for each of the groups tested and their *t* values, along with degrees of freedom (df), standard error of difference, *P*-values and predicted statistical significance are summarized in Supplementary Material, Table S2. Based on the *P*-values the GraphPad Prism software calculates the difference as not significant, not likely to be significant, significant, very significant and extremely significant.

## Supplementary Material

Supplementary Material is available at *HMG* online.

*Conflict of Interest statement.* The authors declare no competing financial interests. 

## Funding 

Actions Concertees Interpasteuriennes (ACIP) and a research grant from the European Society of Pediatric Endocrinology to A.B. A research grant from the EuroDSD in the European Community’s Seventh Framework Programme FP7/2007–2013 under grant agreement No. 201444 as well as grant No. 295097 as part of the EU call FP7-INCO-2011–6 to A.B. and K.McE. A Franco-Egyptian AIRD-STDF grant to A.B., K.M. and I.M. Chinese Government Scholarship and University of the Chinese Academy of Science (UCAS) for financial and infrastructure support to Y.S. 2013 MOST China-EU Science and Technology Cooperation Program, Grant No. 2013DFE33080, by the National Natural Science Foundation of China (Grant No. 31471238) and a 100 talent award of the Chinese Academy of Sciences to R.J. Innovation Fund Denmark (grant # 14–2013-4) to K.A. The human embryonic and fetal material was provided by the Joint MRC/Wellcome Trust (grant # 099175/Z/12/Z) Human Developmental Biology Resource (www.hdbr.org). J.C.A. is a Wellcome Trust Senior Research Fellow in Clinical Science (098513/Z/12/Z) and received support from the National Institute for Health Research Biomedical Research Centre at Great Ormond Street Hospital for Children NHS Foundation Trust and University College London. R.R. is a fellow supported by the Italian Ministry of Health, Rome, Italy (grant # GR-2011–02351636). This work is supported by the COST Action DSDnet BM1303. This work was funded by the Agence Nationale de la Recherche (Laboratoire d’Excellence Revive, Investissement d’Avenir; ANR-10-LABX-73). Funding to pay the Open Access publication charges for this article was provided by Laboratoire d'Excellence Revive, Investissement d'Avenir; ANR-10-LABX-73.

## Supplementary Material

Supplementary Data
