# Supplementary material for: Mutations involving the SRY-related gene SOX8 are associated with a spectrum of human reproductive anomalies
Source: Hum Mol Genet. 2018 Jan 24;27(7):1228–40. doi: 10.1093/hmg/ddy037 (PMC6159538; doi:10.1093/hmg/ddy037)
Supplement: Supplementary Data [file ddy037_sox_si_hmg-2017-d-00942.docx]

**Mutations involving the *SRY*-related gene *SOX8* are associated with a spectrum of human reproductive anomalies**

**Portnoi et al.**

**SUPPLEMENTAL INFORMATION**

**SI METHODS**

**Extended Clinical data - Patient 2**

Patient 2 was born to a 32 year-old G3P0222 mother. At 28 weeks of gestation, fetal ultrasound revealed hydrops, ascites, and cardiomegaly, which led to referral for evaluation of severe fetal anemia. Doppler examination of the middle cerebral artery showed fetal hemoglobin of 4.2%, which was considered to be within a severe range and treated with an intrauterine transfusion of 125 mL of PRBCs. Labor was induced due to concerns for recurrent prenatal anemia at 35 weeks and 2 days weeks of gestation. At birth, the baby weighed 2.43 kg with a length of 46 cm, OFC 34 cm. The baby was considered to be a girl based on initial examination of the external genitalia. Her Apgar scores were 4 at 1 minute and 9 at 5 minutes. An echocardiogram showed a closed PDA with normal heart function. The patient required a short course of phototherapy and was discharged on day 10 of life. A week later the baby was admitted to the NICU for persistent severe post-natal anemia with hemoglobin 7.4 g/dL (normal range:15-20g/dl), hematocrit 21.9%, high reticulocyte count of 2.8% (normal range: <0.1-1.2%), sleepiness, poor feeding and tachycardia. She received 30ml/kg packed RBCs transfusion. On physical exam, she had prominent forehead. Ophthalmological evaluation revealed small bilateral chorioretinal colobomas.

Although the baby was identified as female at birth her genitalia appeared to have ambiguous features including a midline clitorophallic structure less than 1 cm in width and approximately 1 cm in length with a urethral meatus located at the base of the midline structure. Bilateral labioscrotal folds were present with mild rugae and hyperpigmentation. The labioscrotal folds were fused at the midline and gonads were palpable bilaterally within these folds about 1 mL in volume each. There was no uterus or vagina noted, gonads and epididymides were identified on scrotal ultrasound. The right gonad measured 1.2 x 0.6 x 0.8 cm and the left gonad measured 1.1 x 0.4 x 0.7 cm. An abdominal ultrasound showed scattered punctate calcifications in the liver without notable liver ductal dilation, bilateral adrenal glands showing echogenicity in the medulla, mild left renal pelviectasis and patent hepatic vessels. Karyotype analysis showed 46,XY chromosome complement. Laboratory investigations were performed to evaluate the male undervirilization and showed a baseline testosterone of 138 ng/dL (normal 60-400 ng/dl), dihydrotestosterone 36 ng/dL (normal is 12-85 between 30 and 60 days of life), FSH of 2.31 mIU/mL (normal 0.16-4.1 mIU/ml) and LH of 17.35 mIU/mL (normal 0.02-7.0 mIU/ml). Pituitary hormone levels were within normal limits including a TSH of 1.566 uIU/mL, a free T4 of 1.38 ng/dL, a total T4 of 11.5 ug/dL, prolactin of 95.4 ng/mL, ACTH of 13 pg/mL, cortisol 4.4 ug/dL, and growth hormone of 7.31 ng/mL excluding hypopituitarism. Following multiple discussions with the parents and review of available data (XY karyotype, palpable gonads, and testosterone and DHT concentrations), the baby’s sex of rearing was reassigned to male at 2 months of age.

Clinical examination at 3 years and 3 months of age showed mild facial asymmetry and a triangular face with right ear more posteriorly placed as compared to left. He had mild plagiocephaly, prominent occiput, bitemporal narrowing, full forehead, straight anterior hairline, and mild metopic ridging. His weight was 13.2 kg (16th percentile) and height was 92.1 cm (12th percentile). He had pectus excavatum in the lower part and carinatum in the upper part of the chest and short sternum. He had mild developmental delay, vocalized only a few comprehensible words, started to walk at age of 18-19 months, had a wide-based gait with concerns for leg length asymmetry. His blood studies were consistent with mild thalassemia. By that time the patient underwent surgery for hypospadias and chordee repair, posterior urethral mobilization, and scrotoplasty. He had a retracted and very short phallus. Both testes were small and palpable in the inguinal canal. Gonadal histology showed finely packed seminiferous tubules/cords covered focally by a thick, well collagenized tunica albuginea. The seminiferous cords were normal size with somewhat irregular contours and focal increased tortuosity. Germ cells were present in a subset of the seminiferous cords. The intervening stroma was mildly fibrotic with Leydig cells identified by Calretinin staining. An Oct4 stain was negative, showing an absence of germ cells. A SOX9 stain highlighted most of the Sertoli cells with a nuclear staining pattern. An inhibin stain highlighted Sertoli cells also with granular cytoplasmic staining pattern.

**Chromosome analyses.** Karyotyping was performed using standard methods and chromosomes were observed after G and R banding. FISH analysis was carried out using FITC or rhodamin labeled probes localized in the chromosomal breakpoints regions. For the array-CGH, genome wide copy number analysis was performed using Illumina CytoSNP12 BeadChip arrays (Illumina, San Diego, California, USA). The samples were processed using the Infinium assay and results analyzed by Illumina Genome Studio software. Data were analyzed by examining signal intensity (Log R ratio) and allelic composition (B Allele Frequency BAF) with GenomeStudio V2011.1 and CNV Partition Plug-in V.2.4.4 for Genome Studio with the default confidence threshold of 35.

**Immunohistochemistry:** Human fetal testis tissue (9 weeks post conception) was provided with approval from the Human Developmental Biology Resource (HDBR, [www.hdbr.org](http://www.hdbr.org)). Term fetal (40 weeks) ovary was obtained from Abcam (#ab4412) and adult ovary tissue (19 years) was obtained from Tissue Solutions. Tissue sections (12 µm) were fixed briefly in 4% PFA in TBS, rinsed in TBS and blocked in 1% BSA in TBS-Tween (0.5% Tween) for 1 hour before incubating overnight with rabbit monoclonal SOX8 antibody (Sigma HPA41640; 1:200 dilution) and, where relevant, mouse monoclonal NR5A1 (SF1) antibody (Thermo Fisher #434200; 1:200 dilution). Sections were washed in TBS-Tween and incubated for 1 hour with the relevant secondary antibodies: Alexa555 goat anti-rabbit (Invitrogen, A21429; 1:400) and Alexa488 goat anti-mouse (Invitrogen, A11001; 1:400). Nuclei were counterstained with DAPI (Sigma). Slides were washed and mounted using ProLong Gold Antifade Mountant (Lifetech, #P36930). Images were collected on a Zeiss LSM 710 confocal microscope (Carl Zeiss) and analyzed using Zeiss Zen 2009 and Image J.

**Whole Exome sequencing.** For the POI replication study, the whole exome sequencing was performed using Illumina Hiseq 25000 on a cohort of 104 females of European descent affected by primary ovarian insufficiency. The study was approved by the University of Pittsburgh Institutional Review Board (PRO09080427, PRO12120172). Data were aligned to the Human Genome Assembly (hg19) using Burrows-Wheeler Aligner's Smith-Waterman Alignment (BWA) algorithm, version 0.7.3a MEM (Maximum Exactly Match, PMID: 20080505). The Genome Analysis Toolkit (GATK) version 2.5-2 was used for a local realignment near indels, reads base quality recalibration, and variants calling. Variants were filtered for quality, using the GATK suggested hard filter criteria. The remaining variants were annotated using ANNOVAR (Last Change Date: 2013-02-11). Variants that affect exons or splice sites, are non-synonymous changes, and have an allele frequency of less than 1% in 1000 Genomes database and/or the ESP6500 database from NHLBI were further evaluated.

For patient 1, the precise breakpoints were mapped by WGS using NexSeq500 (Illumina) at a average coverage of 13.5. The breakpoints were determined using the Breakdancer software. Breakpoints were confirmed by PCR and Sanger sequencing on DNA from the patient.

110 patients presenting with 46,XY DSD were sequenced using the whole exome approach. Exon enrichment was performed with Agilent SureSelect Human All Exon V4. Paired-end sequencing was performed on the Illumina HiSeq2000 platform with TruSeq v3 chemistry. Read files (fastq) were generated from the sequencing platform via the manufacturer’s proprietary software. Reads were mapped with the Burrows-Wheeler Aligner, and local realignment of the mapped reads around potential insertion/deletion (indel) sites was carried out with GATK version 1.6. Duplicate reads were marked with Picard version 1.62 (http://broadinstitute.github.io/picard/). Additional BAM file manipulations were performed with Samtools (0.1.18). SNP and indel variants were called with the GATK Unified Genotyper for each sample. SNP novelty was determined against dbSNP138. Novel variants were analyzed by a range of web-based bioinformatics tools with the EnsEMBL SNP Effect Predictor (http://www.ensembl.org/homosapiens/userdata/uploadvariations). All variants were screened manually against the Human Gene Mutation Database Professional Biobase (http://www.biobase-international.com/product/hgmd/). *In silico* analysis was performed to determine the potential pathogenicity of the variants. Potentially pathogenic mutations were verified with classic Sanger sequencing.

**Sanger Sequencing of the entire coding sequence of *SOX8*.** All infertile male patients (n=274), 153 women with POI and all in-house control samples were screened for mutations in the *SOX8* gene by direct sequencing. The coding exons of the *SOX8* gene were amplified from DNA extracted using conventional techniques from peripheral blood lymphocytes of each individual. We designed 5 pairs of primers to amplify by PCR the *SOX8* coding exons. Exon 1 amplicon 1, F1: 5’-CGCGGAGCTTTCTTTATGG-3’, R1: 5’-GACTCCAGTCGTAGCCCTTG-3’. Exon 1 amplicon 2, F2: 5-CCCGATGCTGGACATGAG-3’, R2: 5’-CTCGCAGTCCGAGACCAG-3’. Exon 2, F3: 5’-AGAGGAGTGTACTGCCTGGTG-3’, R3: 5’-CACAGCTGCCTCCCATACAG-3’. Exon 3 amplicon 1, F4: 5’-GCGCTTCATGGAATTTTCTC-3’, R4: 5’-GCTGCTCCGTCTTGATGTG-3’. Exon 3 amplicon 2, F5: 5’-CCACGAGTTCGACCAGTACC-3’, R5: 5’-ACTTCAGCAGGCACTTGGAG-3’. The PCR amplification conditions were, F1/R1, F2/R2 and F3/R3 - 95°C for 5 min followed by 37 cycles of 98°C 30s, 60°C 30s, 72°C 30s. For the amplicons F4/R4 and F5/R5 the annealing temperature was 65°C. PCR fragments (5 µl) were then electrophoresed in a 2% agarose gel stained in ethidium bromide (1 µg/mL) to verify the expected length of the amplified fragments. DNA sequence analysis was performed using at least 200 ng of purified DNA, 20 ng of primer and fluorescently labeled Taq DyeDeoxy terminator reaction mix (Applied Biosystems) according to the manufacturer’s instructions. DNA sequence was determined using an ABI 3700 automated DNA sequencer.

**Plasmid construction.** Vector containing full-length human *SOX8* in a pCMV6-AN-Myc vector was purchased from Origene (<http://www.origene.com/>). The pCMX-*NR5A1* vector with human *NR5A1*, and *AMH* as well as *Tesco* reporters have been previously described [1]. The pCDNA-*SOX9*-Flag vector was a gift from Dr. Francis Poulat ([Institut de Génétique Moléculaire de Montpellier](http://www.researchgate.net/institution/Institut_de_Genetique_Moleculaire_de_Montpellier), France). The reporter construct with mouse *Dmrt1* promoter was a gift from Dr. David Zarkower (Univ. of Minnesota, USA).

**Site-Directed Mutagenesis.** *SOX8* expression vectors containing the variants were generated by site-directed mutagenesis (QuikChange, Stratagene) with the use of wild-type (WT) human *SOX8* cDNA in pCMV6-AN-Myc expression vector as a template. The entire coding sequence of all mutant plasmids was confirmed by direct sequencing prior to further studies.

**Cell lines, transfections and protein preparation.** HEK293-T cells were a gift from Mlle Aurélie Claes (Institut Pasteur). For all the in-vitro assays either HEK293-T or E14 mES cells were transfected using FuGENE 6 (Roche) transfection reagent.

Whole cellular protein was extracted using IP Lysis Buffer (Pierce). Briefly, the culture medium was removed from the wells and washed once with ice-cold 1x PBS. Then, 500 µl of ice-cold lysis buffer was added to the cells and further incubated on ice for 5 mins with periodic mixing. Lysate was transferred to a microcentrifuge tube and centrifuged at ~13,000g for 10 minutes at 4°C to pellet the cell debris. Supernatant was assayed for total protein concentration using a Coomassie Plus (Bradford) Protein Assay (Pierce). The efficiency of transfection and presence of specific proteins was detected by western blot analysis using anti-SOX8 (mouse, #ab57197, Abcam®), anti-NR5A1 (rabbit, #ab65815, Abcam®) or anti-SOX9 (rabbit, #ab59265, Abcam®) antibodies.

**Structural Modeling.** Binary SOXE/DNA complexes were generated with I-TASSER using human SOX8 (Uniprot = P57073) and SOX9 (Uniprot = P48436) sequences and the mouse SOX17/DNA complex (PDBID = 3F27) as a template [2, 3]. For the ternary SOX8–OCT4-DNA complex a previously generated SOX2–OCT4 model on the canonical SOX-OCT DNA element (GGCATTGTCATGCAAATCGGCGG) was used as a template [4]. The ternary complexes of SOX8E156D–SOX9, SOX8E156D–SOX8 were generated using chimera 1.10.1 (<http://www.cgl.ucsf.edu/chimera/>) by superimposing DNA fragments to modeled ideal B-DNA corresponding to a palindromic SOXE consensus sequence with a 3 base-pair spacer between the SOX elements (CCCGGACAATGAAACATTGTCCGGG, base-pairs used for superposition are underlined and the core SOX motifs are shown in italics) [5]. Next, sequences of the original *Lama1* DNA in 3F27 were converted into corresponding nucleotides in the ideal sequence and overlapping nucleotides were deleted. The phosphodiester bonds between juxtaposed SOX-E/DNA complexes were created using the chimera ‘adjust bond’ option. The energy of the ternary complex models was minimized in chimera by using amber force fields ff14SB for the protein and Bsc0 for DNA. The final models were validated using the w3DNA server and the integrity of protein-DNA contacts was checked using nucplot [6, 7]. All figures were generated using chimera.

**Cellular Localization.** Cellular localization of both WT-SOX8 and mutants were assayed by transfecting the different plasmids into HEK 293-T cells with the use of FuGENE 6 (Roche) in chamber slides (Nunc). Cells were fixed in 3-4% paraformaldehyde in PBS (pH 7.4) for 15 min at room temperature, 48 hrs post transfection, washed twice with ice cold 1xPBS, permeabilized in 1xPBS containing 0.25% Triton X-100, and then incubated with 1% BSA in PBS-T for 30 min to block non-specific binding of the antibodies. Cells were incubated with the primary antibody (diluted in 1% BSA in PBS-T) in a humid chamber overnight at +4°C. The following dilutions of primary antibodies were used: anti-Myc (rabbit, #ab9106, Abcam®), 1:100 and anti-NR5A1 (rabbit, #ab65815, Abcam®), 1:100. After 16 h incubation, cells were washed three times with 1xPBS for 5 min each. This was followed by incubation with the secondary antibody in 1% BSA for 1h at room temperature in the dark Goat anti-Rabbit IgG (H+L) Secondary Antibody, Alexa Fluor® 488 conjugate, #A11034, and Goat anti-Rabbit IgG (H+L) Secondary Antibody, Alexa Fluor® 594 conjugate, Life Technologies, both diluted to 1:1000), cells were washed three times with 1xPBS for 5 min each in the dark. The coverslips were mounted with a drop of mounting medium containing DAPI (DNA stain) for 1 min and sealed with nail varnish to prevent drying and movement. Images were obtained with a Leica Microsystems DMI4000B microscope at 40x or 63x magnifications.

**Transient Gene Expression Assays.** Transient gene expression assays to assess SOX8 function were performed in 96-well plates (Nunc) using either HEK293-T human embryonic kidney cells or mouse ES (mES) cell line, E14, FuGENE 6 and a Dual-Luciferase reporter assay system (Promega) with pRL-CMV Renilla luciferase (Promega) expression as a marker of transfection efficiency as described previously^1^. pCMV6-*SOX8-*Myc WT or mutant expression vectors (10 ng/well) were co-transfected with or without pCMX-*NR5A1* or pCDNA-*SOX9*-Flag into HEK293-T or mES cells with reporter vectors and vector containing Renilla luciferase (10 ng/well). Cells were lysed 48 hours later and luciferase assays were performed (Dual Luciferase Reporter Assay system, Promega) using a Centrox3 LB960 (Berthold Technologies). All data were standardized for Renilla luciferase activity. Results are shown as the mean ± SEM of at least three independent experiments, each performed at least in quadruplicate.

**Co-Immunoprecipitation assay.** Whole cell lysate was prepared from the HEK293-T human embryonic kidney cells in 6-well plates (TPP) transfected using FuGENE 6. The cells were transfected with pCMV6-*SOX8*-Myc WT or mutant expression vectors (1 µg/well) with or without pCMX-*NR5A1* or pCDNA-*SOX9*. Transfection and protein extraction are described above. Total cellular protein extract (250 μg) was pre-cleared by adding 20 μl of resuspended Protein A/G PLUS-Agarose (Santa Cruz Biotechnology, sc2003) and 5 μl of normal mouse IgG (Santa Cruz Biotechnology, sc2025) followed by incubation at 4°C on a rocker platform for 1 hour. The mix was centrifuged at 1,000g for 5 mins at 4°C and supernatant containing pre-cleared protein was collected in a new tube. The pre-cleared lysate was incubated overnight on a rocker platform at 4°C with 50 ng either anti-SOX8 (mouse, #ab57197, Abcam®), anti-SOX9 (rabbit, #ab59265, Abcam®) or anti-NR5A1 (rabbit, #ab65815, Abcam®) antibodies as well as 25 μl of resuspended Protein A/G PLUS-Agarose. The antibody-protein-bead complex was centrifuged at 1,000g for 5 mins at 4°C and the supernatant discarded. The pellet was washed 4 times with 1 ml ice-cold 1xPBS and finally resuspended in 20 μl of Laemmeli buffer (Biorad). The resuspended protein was boiled for 2 mins and 25 μl was migrated on a 5% SDS-PAGE gel and transferred to nitrocellulose (PVDF) membrane (Amersham). Proteins were detected with anti-NR5A1 (rabbit, #ab65815, Abcam®) Abcam), anti-SOX9 (rabbit, #ab59265, Abcam®) or anti-SOX8 (mouse, #ab57197, Abcam®) antibodies, followed by secondary antibody conjugated with horseradish peroxidase and visualized by chemiluminiscence using ECL Western blotting Substrate (Pierce).

**Duolink proximity ligation assay.** Protein-protein interaction was detected by Duolink proximity ligation assay (PLA) Kit (Sigma-Aldrich: PLA Probe anti-rabbit plus; PLA Probe anti-mouse minus; PLA Probe anti-goat minus, Detection Kit DUO92014). The PLA probe anti-rabbit plus binds to the SOX9 and SF1 antibodies (ABCAM), whereas the PLA probe anti-mouse minus binds to the SOX8 antibody (ABCAM). If the distance between both proteins is <40 nm, a signal with DuoLink PLA is generated, thus indicating an interaction of both proteins [8]. After pre-incubation with blocking agent for 1 h, transfected HEK cells were incubated 2 hours at room temperature with the primary anti-SOX9 (1/100) and anti-SF1 (1/100) antibodies then overnight with the primary anti-SOX8 antibody (1:100). Duolink PLA probes detecting rabbit or mouse antibodies were diluted in the blocking agent to a concentration of 1:5 and applied to the slides, followed by incubation for 1 hour in a humidity chamber at 37°C. Unbound PLA probes were removed by washing. For ligation of the two Duolink PLA probes, Duolink ligation stock (dilution 1:5) was used. The cells were incubated in the ligation solution consisting of Duolink Ligation stock (1:5) and Duolink Ligase (1:40) for 30 min at 37°C. Detection of the amplified probe was done with the Duolink Detection Kit. Duolink Detection stock was diluted at 1:5 and applied for 100 min at 37°C. Slides were then mounted using ProLong® Gold Antifade Mountant with DAPI.

**SI - SUPPLEMENTARY REFERENCES**

1. Bashamboo A, Brauner R, Bignon-Topalovic J, Lortat-Jacob S, Karageorgou V, Lourenco D, Guffanti A, McElreavey K (2014) Mutations in the FOG2/ZFPM2 gene are associated with anomalies of human testis determination. *Hum Mol Genet*., 23, 3657-3665.

2. Yang J, Yan R, Roy A, Xu D, Poisson J, Zhang Y (2015) The I-TASSER Suite: protein structure and function prediction. *Nat Methods*., 12, 7-8.

3. Palasingam P, Jauch R, Ng CK, Kolatkar PR. (2009) The structure of Sox17 bound to DNA reveals a conserved bending topology but selective protein interaction platforms. *J Mol Biol*., 388, 619-630.

4. Merino F, Ng CK, Veerapandian V, Schöler HR, Jauch R, Cojocaru V. (2014) Structural basis for the SOX-dependent genomic redistribution of OCT4 in stem cell differentiation. *Structure*., 22, 1274-1286.

5. Pettersen EF, Goddard TD, Huang CC, Couch GS, Greenblatt DM, Meng EC, Ferrin TE. (2004) UCSF Chimera--a visualization system for exploratory research and analysis. *J Comput Chem.,* 25, 1605-1612.

6. Zheng G, Lu XJ, Olson WK. (2009) Web 3DNA--a web server for the analysis, reconstruction, and visualization of three-dimensional nucleic-acid structures. *Nucleic Acids Res*., 37, W240-246.

7. Luscombe NM, Laskowski RA, Thornton JM. (1997) NUCPLOT: a program to generate schematic diagrams of protein-nucleic acid interactions. *Nucleic Acids Res.*, 25, 4940-4945.

8. Söderberg O, Leuchowius KJ, Gullberg M, Jarvius M, Weibrecht I, Larsson LG, Landegren U. (2008) Characterizing proteins and their interactions in cells and tissues using the in situ proximity ligation assay. *Methods.*, 45, 227-232.

9. Uhlén M, Fagerberg L, Hallström BM, Lindskog C, Oksvold P, Mardinoglu A, Sivertsson Å, Kampf C, Sjöstedt E, Asplund A et al. Proteomics. Tissue-based map of the human proteome. *Science*. 2015;347:1260419.

**Mutations involving the *SRY*-related gene *SOX8* are associated with a spectrum of human reproductive anomalies**

**Portnoi et al.**

**SUPPLEMENTAL INFORMATION**

**SI FIGURES**

**Supplementary Figure 1. Expression of SOX8 in murine and human gonadal tissues.** (A) Murine *Sox8* expression by in situ hybridization in the developing testis cords at 15.5 d.p.c. (GUDMAP:6637, www.gudmap.org). (B) SOX8 protein expression in human normal 46 yr old male testis. Expression is confined to Leydig cells (L), Sertoli cells (S) and spermatocytes (Sp). Bar 100 μm ([www.proteinatlas.org](http://www.proteinatlas.org);9) (C) SOX8 expression in normal 39 yr old ovary. Antral follicle showing high SOX8 expression in the granulosa cells (G) and lower expression in theca layers (T) and stroma (S) ([www.proteinatlas.org](http://www.proteinatlas.org)). Bar 100 μm ([www.proteinatlas.org](http://www.proteinatlas.org); 9).

**Supplementary Figure 2.** **Protein-protein interaction of SOX8-WT and SOX8p.Glu156Asp with NR5A1 and SOX9.** Plasmids encoding SOX8-WT or SOX8p.Glu156Asp were transiently expressed with WT-NR5A1 or SOX9 for 48hrs in HEK293T cells. SOX8-NR5A1 or SOX8-NR5A1 complexes were immunoprecipitated from total protein extracts using A/G agarose beads and an antibody specific for NR5A1 or SOX9; the bound SOX8 protein was detected by western blot using an anti-SOX8 antibody followed by secondary antibody conjugated with HRP. Both the SOX8-WT and SOX8p.Glu156Asp proteins can physically interact with NR5A1. Whereas the SOX8-WT protein can interact with SOX9, the mutant SOX8p.Glu156Asp cannot.

**Supplementary Figure 3.** **Dominant negative effect of SOX8p.Gly156Asp on synergy between NR5A1 and wild-type SOX8.** HEK293-T cells were transfected with *Tesco* enhancer reporter (10ng), 1 ng of NR5A1+ 1 ng of SOX8p.Gly156Asp vector with increasing amounts of wild-type SOX8 expression vector (0, 1, 2, 5, 10 ng). Increasing amounts of mutant SOX8p.Gly156Asp expression vector (0, 1, 2, 5, 10 ng) was also co-transfected with 1 ng of wild-type SOX8 + 1ng of NR5A1 and *Tesco* enhancer (10ng). pRL-CMV Renilla luciferase vector was co-transfected as a marker of transfection efficiency. Results are expressed as the RLU. The SOX8p.Gly156Asp vector exhibits dominant negative activity on synergistic activation of *Tesco* enhancer by NR5A1 and wild-type-SOX8 even at 2 times higher concentrations.

**Supplementary Figure 4.** **Transcriptional activities of mutant SOX8 proteins.** The transcriptional activities of *SOX8-*WT and mutants were studied using the human *AMH* and *NR5A1*and mouse *Dmrt1* promoters as reporters following transfection in HEK293-T or mouse ES cells. The data shown here represent the mean ± SEM of minimum three independent experiments, each of which was performed at least in quadruplicate. The reporter constructs were transfected into cells with either the WT-*SOX8* or mutants expression vectors. The results are expressed as relative percentage of WT-*SOX8* activity (100%). Data are shown for mutants associated with male infertility (blue) or female infertility (red). The SOX8p.D382N mutation was observed in both male and female infertility. Mutants showed altered biological activity compared with the WT protein. The position and alignment of the mutants associated with 46,XY individuals (blue), 46,XX individuals (red), and both 46,XY and 46,XX individuals (green) are shown. The blue box corresponds to DIM domain, the pink box to the HMG domain and the green boxes represent the transactivation domains of the SOX8 proteins.

**Supplementary Figure 5. Cellular localisation of mutant SOX8 proteins** (A) Human embryonic kidney (HEK 293-T) cells were transfected with myc-tagged WT-SOX8 or SOX8p.Glu156Asp with untagged NR5A1 expression vectors. 48 hours post transfection the cells were fixed and the co-expression of WT-SOX8 and SOX8p.Glu156A along with NR5A1 was detected using anti-Myc (green) and anti-SF1 (red) antibodies respectively. The nucleus is stained by DAPI (blue). When co-transfected both WT-SOX8 and SOX8p.Glu156Asp co-localize to the nucleus. However, when transfected alone SOX8p.Glu156Asp shows an equal distribution between the nucleus and the cytoplasm, as compared to WT protein, which is mostly nuclear. The distribution was evaluated by counting at least 100 transfected cells each from five independent experiments. Scale bars are 20 µm. Original magnification x40. (B) Human embryonic kidney (HEK 293-T) cells were transfected with myc-tagged WT-*SOX8* or mutants associated with male infertility (p.K241T, p.G378S) or female infertility (p.R8_S9del, S267L). At 48 hours post transfection the cells were fixed and SOX8 was detected using anti-Myc antibody (green). The nucleus is stained with DAPI (blue). The mutant SOX8 proteins show variable distribution between the nucleus and the cytoplasm, as compared to WT protein, which is mostly nuclear (70 %). The distribution was evaluated by counting at least 100 transfected cells each from five independent experiments. Scale bars are 20 µm. Original magnification x40.

Supplemental Figure 1


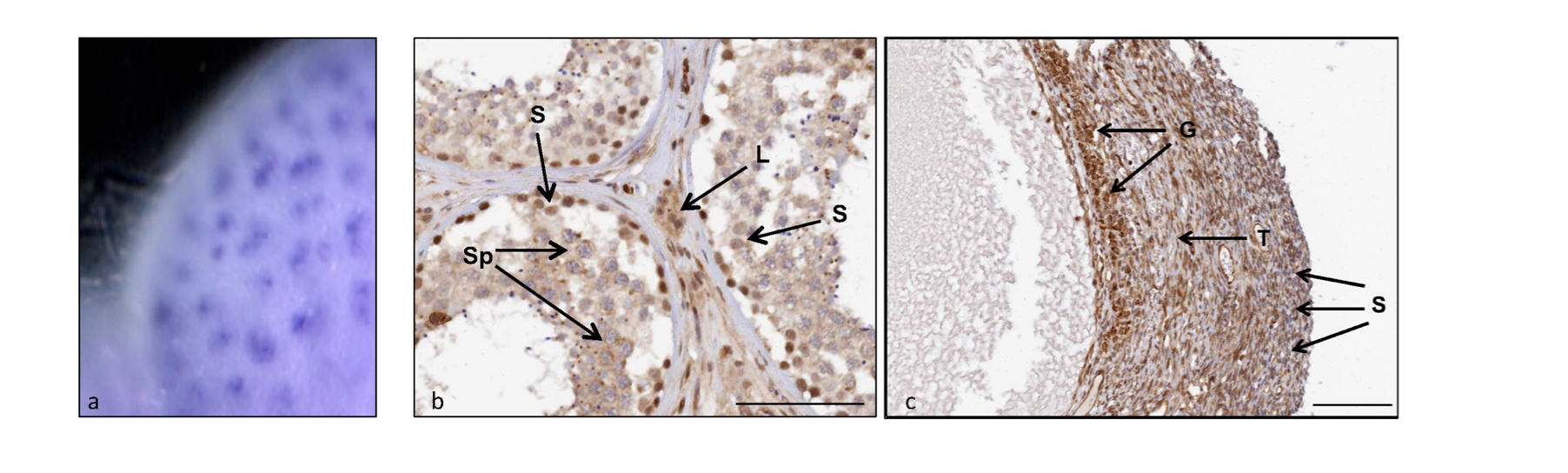


Supplemental Figure 2


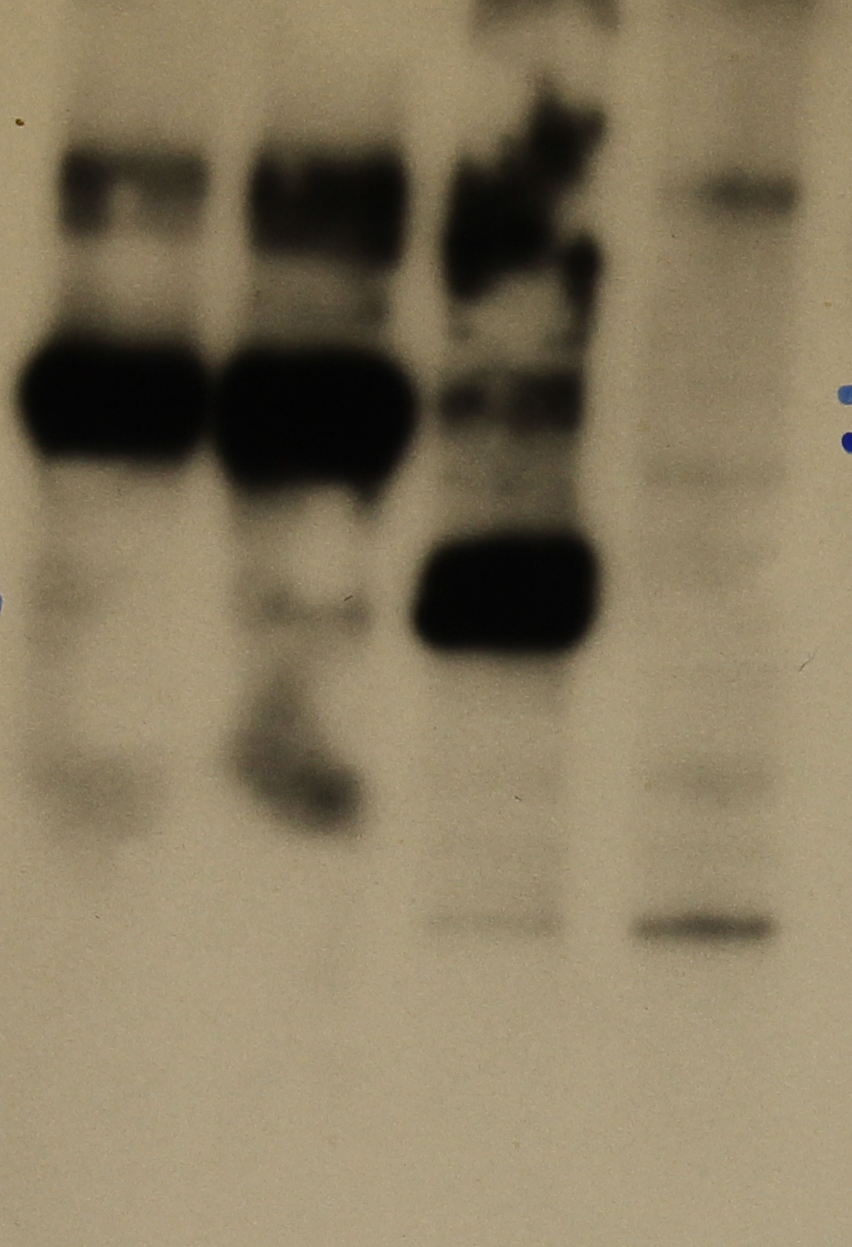


**NR5A1 + + - -**

**SOX8-WT + - + -**

**SOX8-E156D - + - -**

**NR5A1**

**+SOX8**

**SOX8**

A


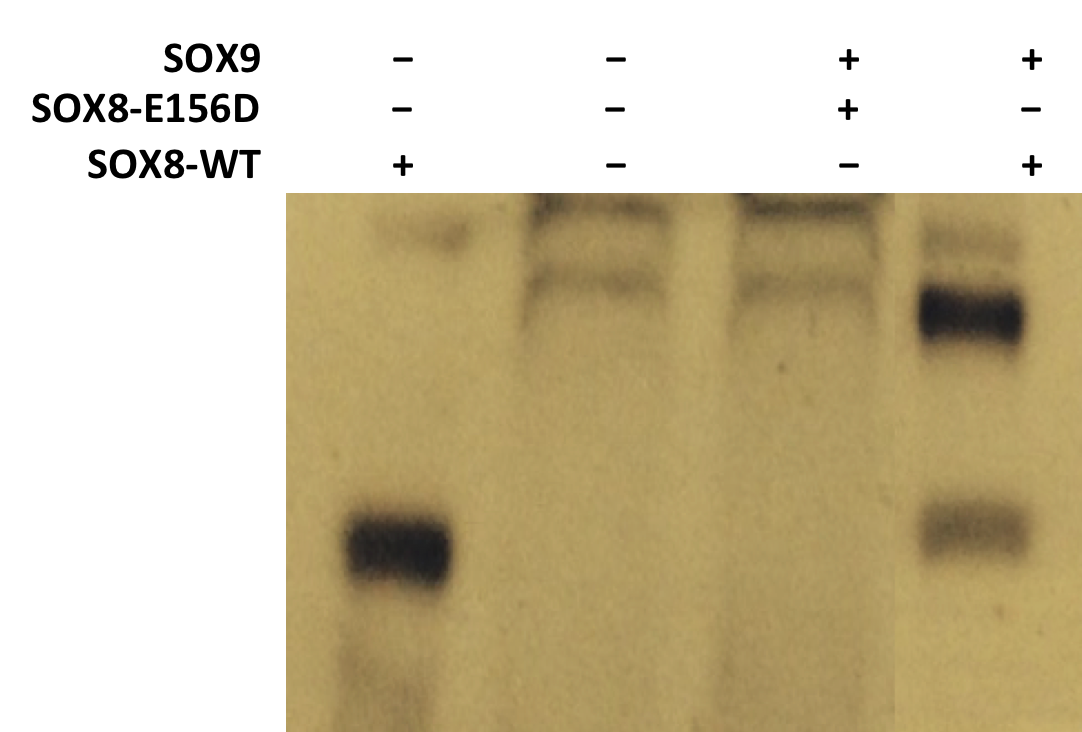


**SOX8**

B

Supplemental Figure 3


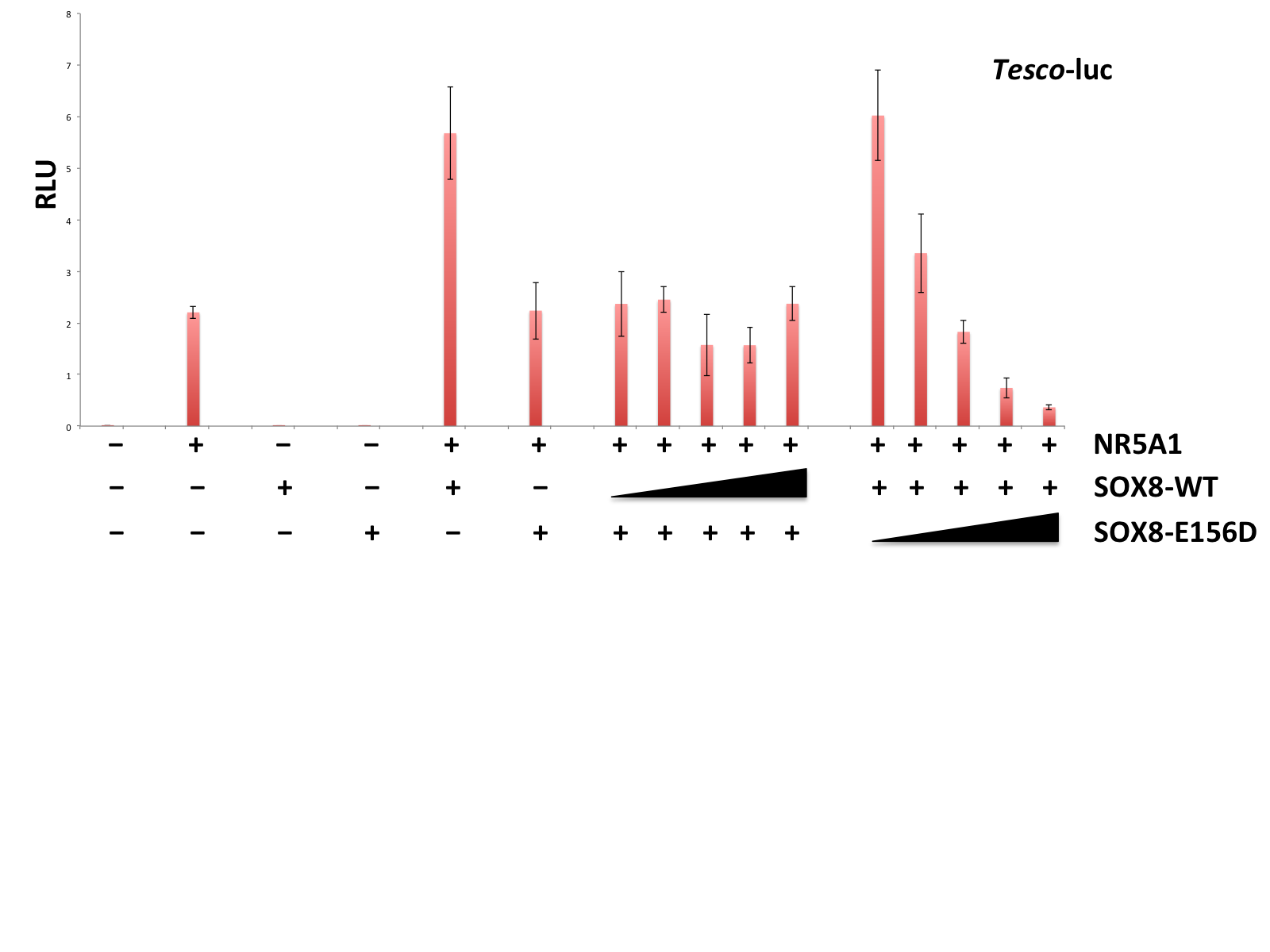


Supplemental Figure 4


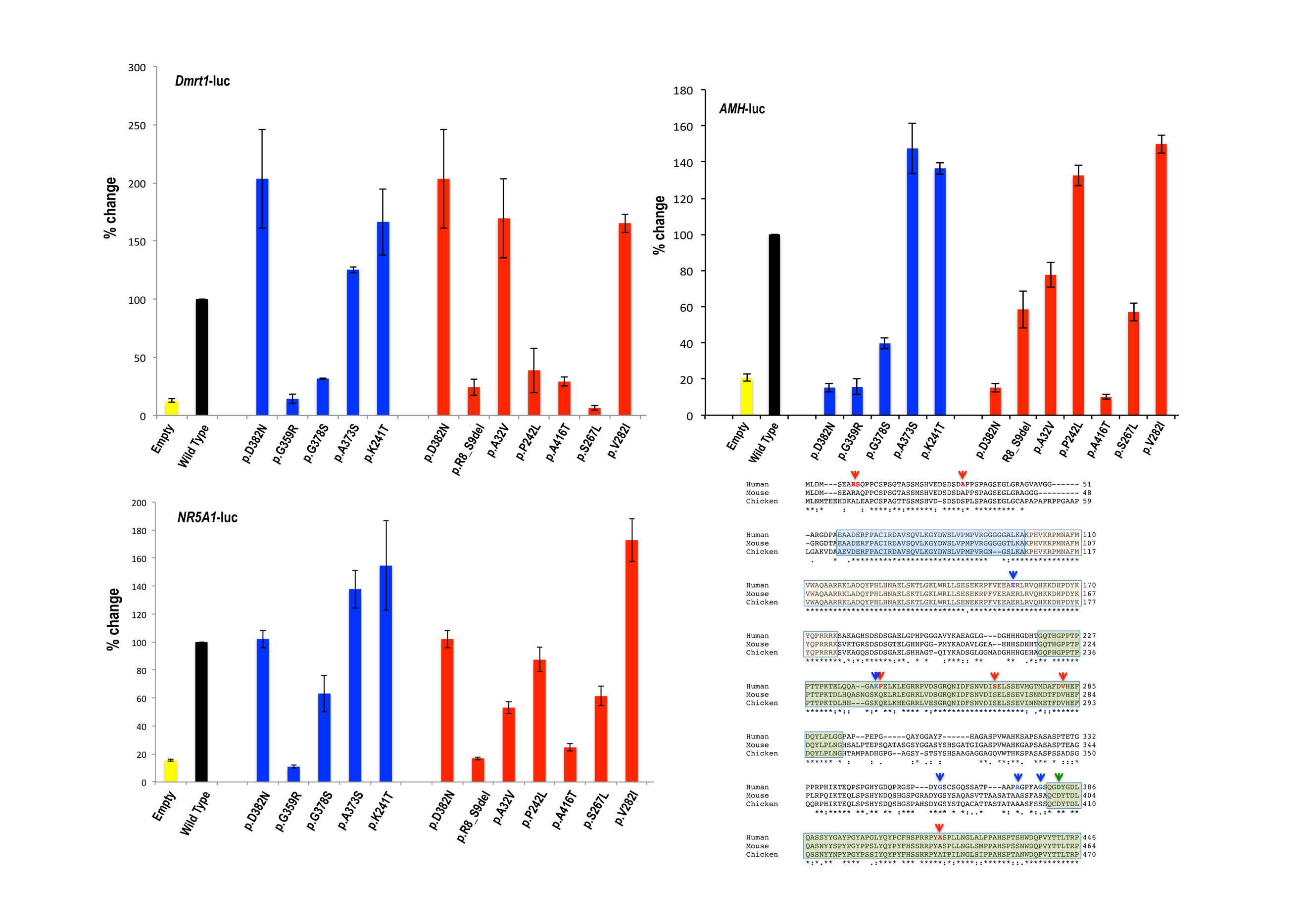


Supplemental Figure 5

A

B


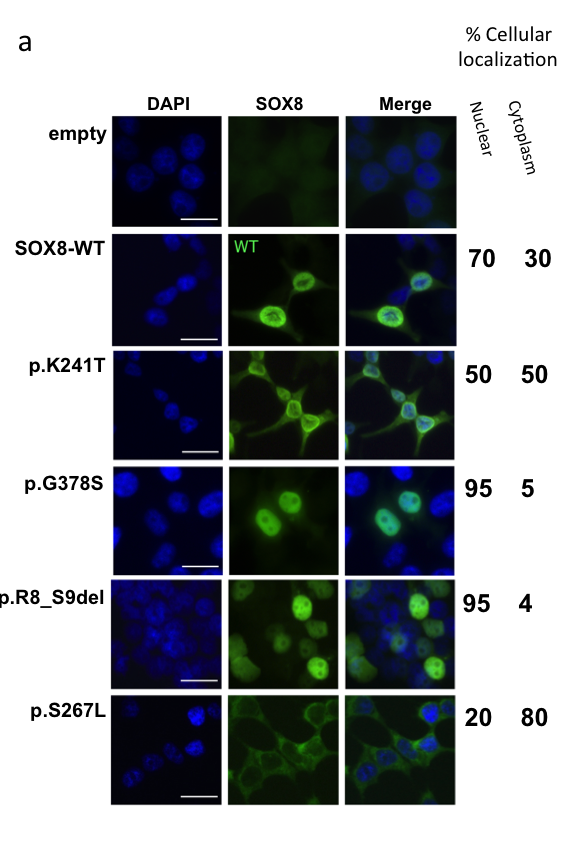


| **Gene** | **Entrez Gene Summary** | **UniProt Disorders Summary** |
| --- | --- | --- |
| *NPRL3* | The function of the encoded protein is not known. | Inactivating mutations and truncating deletions in the genes encoding GATOR1 proteins are detected in glioblastoma and ovarian tumors and are associated with loss of heterozygosity events. Inactivation of GATOR1 proteins promotes constitutive localization of mTORC1 to the lysosomal membrane and blocks mTORC1 inactivation following amino acid withdrawal (PubMed:23723238). |
| *HBZ* | Zeta-globin is an alpha-like hemoglobin. The zeta-globin polypeptide is synthesized in the yolk sac of the early embryo, while alpha-globin is produced throughout fetal and adult life. The zeta-globin gene is a member of the human alpha-globin gene cluster that includes five functional genes and two pseudogenes. The order of genes is: 5' - zeta - pseudozeta - mu - pseudoalpha-1 - alpha-2 -alpha-1 - theta1 - 3'. | **-** |
| *HBM* | The human alpha globin gene cluster located on chromosome 16 spans about 30 kb and includes seven loci: 5'- zeta - pseudozeta - mu - pseudoalpha-1 - alpha-2 - alpha-1 - theta - 3'. This gene has an ORF encoding a 141 aa polypeptide which is similar to the delta globins found in reptiles and birds. | **-** |
| *HBA2* | The human alpha globin gene cluster located on chromosome 16 spans about 30 kb and includes seven loci: 5'- zeta - pseudozeta - mu - pseudoalpha-1 - alpha-2 - alpha-1 - theta - 3'. The alpha-2 (HBA2) and alpha-1 (HBA1) coding sequences are identical. These genes differ slightly over the 5' untranslated regions and the introns, but they differ significantly over the 3' untranslated regions. Two alpha chains plus two beta chains constitute HbA, which in normal adult life comprises about 97% of the total hemoglobin; alpha chains combine with delta chains to constitute HbA-2, which with HbF (fetal hemoglobin) makes up the remaining 3% of adult hemoglobin. Alpha thalassemias result from deletions of each of the alpha genes as well as deletions of both HBA2 and HBA1; some nondeletion alpha thalassemias have also been reported. | Alpha-thalassemia (A-THAL) [MIM:604131]: A form of thalassemia. Thalassemias are common monogenic diseases occurring mostly in Mediterranean and Southeast Asian populations. The hallmark of alpha-thalassemia is an imbalance in globin-chain production in the adult HbA molecule. The level of alpha chain production can range from none to very nearly normal levels. Deletion of both copies of each of the two alpha-globin genes causes alpha(0)-thalassemia, also known as homozygous alpha thalassemia. Due to the complete absence of alpha chains, the predominant fetal hemoglobin is a tetramer of gamma-chains (Bart hemoglobin) that has essentially no oxygen carrying capacity. This causes oxygen starvation in the fetal tissues leading to prenatal lethality or early neonatal death. The loss of two alpha genes results in mild alpha-thalassemia, also known as heterozygous alpha-thalassemia. Affected individuals have small red cells and a mild anemia (microcytosis). If three of the four alpha-globin genes are functional, individuals are completely asymptomatic. Some rare forms of alpha-thalassemia are due to point mutations (non-deletional alpha-thalassemia).  Heinz body anemias (HEIBAN) [MIM:140700]: Form of non-spherocytic hemolytic anemia of Dacie type 1. After splenectomy, which has little benefit, basophilic inclusions called Heinz bodies are demonstrable in the erythrocytes. Before splenectomy, diffuse or punctate basophilia may be evident. Most of these cases are probably instances of hemoglobinopathy. The hemoglobin demonstrates heat lability. Heinz bodies are observed also with the Ivemark syndrome (asplenia with cardiovascular anomalies) and with glutathione peroxidase deficiency. {ECO:0000269 PubMed:2833478}.  Hemoglobin H disease (HBH) [MIM:613978]: A form of alpha-thalassemia due to the loss of three alpha genes. This results in high levels of a tetramer of four beta chains (hemoglobin H), causing a severe and life-threatening anemia. Untreated, most patients die in childhood or early adolescence. {ECO:0000269 PubMed:10569720}.  Alpha(0)-thalassemia is associated with non-immune hydrops fetalis, a generalized edema of the fetus with fluid accumulation in the body cavities due to non-immune causes. Non-immune hydrops fetalis is not a diagnosis in itself but a symptom, a feature of many genetic disorders, and the end-stage of a wide variety of disorders. |
| *HBA1* | The human alpha globin gene cluster located on chromosome 16 spans about 30 kb and includes seven loci: 5'- zeta - pseudozeta - mu - pseudoalpha-1 - alpha-2 - alpha-1 - theta - 3'. The alpha-2 (HBA2) and alpha-1 (HBA1) coding sequences are identical. These genes differ slightly over the 5' untranslated regions and the introns, but they differ significantly over the 3' untranslated regions. Two alpha chains plus two beta chains constitute HbA, which in normal adult life comprises about 97% of the total hemoglobin; alpha chains combine with delta chains to constitute HbA-2, which with HbF (fetal hemoglobin) makes up the remaining 3% of adult hemoglobin. Alpha thalassemias result from deletions of each of the alpha genes as well as deletions of both HBA2 and HBA1; some nondeletion alpha thalassemias have also been reported. | \| Alpha-thalassemia (A-THAL) [MIM:604131]: A form of thalassemia. Thalassemias are common monogenic diseases occurring mostly in Mediterranean and Southeast Asian populations. The hallmark of alpha-thalassemia is an imbalance in globin-chain production in the adult HbA molecule. The level of alpha chain production can range from none to very nearly normal levels. Deletion of both copies of each of the two alpha-globin genes causes alpha(0)-thalassemia, also known as homozygous alpha thalassemia. Due to the complete absence of alpha chains, the predominant fetal hemoglobin is a tetramer of gamma-chains (Bart hemoglobin) that has essentially no oxygen carrying capacity. This causes oxygen starvation in the fetal tissues leading to prenatal lethality or early neonatal death. The loss of two alpha genes results in mild alpha-thalassemia, also known as heterozygous alpha-thalassemia. Affected individuals have small red cells and a mild anemia (microcytosis). If three of the four alpha-globin genes are functional, individuals are completely asymptomatic. Some rare forms of alpha-thalassemia are due to point mutations (non-deletional alpha-thalassemia). Note=The disease is caused by mutations affecting the gene represented in this entry. \| \| --- \| \| Heinz body anemias (HEIBAN) [MIM:140700]: Form of non-spherocytic hemolytic anemia of Dacie type 1. After splenectomy, which has little benefit, basophilic inclusions called Heinz bodies are demonstrable in the erythrocytes. Before splenectomy, diffuse or punctate basophilia may be evident. Most of these cases are probably instances of hemoglobinopathy. The hemoglobin demonstrates heat lability. Heinz bodies are observed also with the Ivemark syndrome (asplenia with cardiovascular anomalies) and with glutathione peroxidase deficiency. {ECO:0000269 PubMed:2833478}. \| \| Hemoglobin H disease (HBH) [MIM:613978]: A form of alpha-thalassemia due to the loss of three alpha genes. This results in high levels of a tetramer of four beta chains (hemoglobin H), causing a severe and life-threatening anemia. Untreated, most patients die in childhood or early adolescence. {ECO:0000269 PubMed:10569720}. \| \| NAlpha(0)-thalassemia is associated with non-immune hydrops fetalis, a generalized edema of the fetus with fluid accumulation in the body cavities due to non-immune causes. Non-immune hydrops fetalis is not a diagnosis in itself but a symptom, a feature of many genetic disorders, and the end-stage of a wide variety of disorders. \| |
| *HBQ1* | Theta-globin mRNA is found in human fetal erythroid tissue but not in adult erythroid or other nonerythroid tissue. The theta-1 gene may be expressed very early in embryonic life, perhaps sometime before 5 weeks. Theta-1 is a member of the human alpha-globin gene cluster that involves five functional genes and two pseudogenes. The order of genes is: 5' - zeta - pseudozeta - mu - pseudoalpha-2 -pseudoalpha-1 - alpha-2 - alpha-1 - theta-1 - 3'. Research supports a transcriptionally active role for the gene and a functional role for the peptide in specific cells, possibly those of early erythroid tissue. |  |
| *LUC7L* | The LUC7L gene may represent a mammalian heterochromatic gene, encoding a putative RNA-binding protein similar to the yeast Luc7p subunit of the U1 snRNP splicing complex that is normally required for 5-prime splice site selection (Tufarelli et al., 2001 [PubMed 11170747]) |  |
| *RGS11* | The protein encoded by this gene belongs to the RGS (regulator of G protein signaling) family. Members of the RGS family act as GTPase-activating proteins on the alpha subunits of heterotrimeric, signal-transducing G proteins. This protein inhibits signal transduction by increasing the GTPase activity of G protein alpha subunits, thereby driving them into their inactive GDP-bound form. |  |
| *ARHGDIG* | The GDP-dissociation inhibitors (GDIs) play a primary role in modulating the activation of GTPases by inhibiting the exchange of GDP for GTP. See ARHGDIB (MIM 602843). |  |
| *PDIA2* | Protein disulfide isomerases (EC 5.3.4.1), such as PDIP, are endoplasmic reticulum (ER) resident proteins that catalyze protein folding and thiol-disulfide interchange reactions (Desilva et al., 1996 [PubMed 8561901]). |  |
| *AXIN1* | This gene encodes a cytoplasmic protein which contains a regulation of G-protein signaling (RGS) domain and a dishevelled and axin (DIX) domain. The encoded protein interacts with adenomatosis polyposis coli, catenin beta-1, glycogen synthase kinase 3 beta, protein phosphate 2, and itself. This protein functions as a negative regulator of the wingless-type MMTV integration site family, member 1 (WNT) signaling pathway and can induce apoptosis. The crystal structure of a portion of this protein, alone and in a complex with other proteins, has been resolved. Mutations in this gene have been associated with hepatocellular carcinoma, hepatoblastomas, ovarian endometriod adenocarcinomas, and medullablastomas. Alternative splicing results in multiple transcript variants. | Caudal duplication anomaly (CADUA) [MIM:607864]: A condition characterized by the occurrence of duplications of different organs in the caudal region. {ECO:0000269 PubMed:16773576}..  Hepatocellular carcinoma (HCC) [MIM:114550]: A primary malignant neoplasm of epithelial liver cells. The major risk factors for HCC are chronic hepatitis B virus (HBV) infection, chronic hepatitis C virus (HCV) infection, prolonged dietary aflatoxin exposure, alcoholic cirrhosis, and cirrhosis due to other causes. {ECO:0000269 PubMed:12101426}. |
| *MRPL28* | Mammalian mitochondrial ribosomal proteins are encoded by nuclear genes and help in protein synthesis within the mitochondrion. Mitochondrial ribosomes (mitoribosomes) consist of a small 28S subunit and a large 39S subunit. They have an estimated 75% protein to rRNA composition compared to prokaryotic ribosomes, where this ratio is reversed. Another difference between mammalian mitoribosomes and prokaryotic ribosomes is that the latter contain a 5S rRNA. |  |
| *NME4* | The nucleoside diphosphate (NDP) kinases (EC 2.7.4.6) are ubiquitous enzymes that catalyze transfer of gamma-phosphates, via a phosphohistidine intermediate, between nucleoside and dioxynucleoside tri- and diphosphates. The enzymes are products of the nm23 gene family, which includes NME4 (Milon et al., 1997 [PubMed 9099850]). |  |
| *RAB11FIP3* | Proteins of the large Rab GTPase family (see RAB1A; MIM 179508) have regulatory roles in the formation, targeting, and fusion of intracellular transport vesicles. RAB11FIP3 is one of many proteins that interact with and regulate Rab GTPases (Hales et al., 2001 [PubMed 11495908]). |  |
| *SOLH* | This gene encodes a protein containing zinc-finger-like repeats and a calpain-like protease domain. The encoded protein may function as a transcription factor, RNA-binding protein, or in protein-protein interactions during visual system development. |  |
| *PIGQ* | This gene is involved in the first step in glycosylphosphatidylinositol (GPI)-anchor biosynthesis. The GPI-anchor is a glycolipid found on many blood cells and serves to anchor proteins to the cell surface. This gene encodes a N-acetylglucosaminyl transferase component that is part of the complex that catalyzes transfer of N-acetylglucosamine (GlcNAc) from UDP-GlcNAc to phosphatidylinositol (PI). |  |
| *WFIKKN1* | This gene encodes a secreted multidomain protein consisting of a signal peptide, a WAP domain, a follistatin domain, an immunoglobulin domain, two tandem Kunitz domains, and an NTR domain. These domains have been implicated frequently in inhibition of various types of proteases, suggesting that the encoded protein may be a multivalent protease inhibitor and may control the action of multiple types of serine proteases as well as metalloproteinases. |  |
| *RHOT2* | This gene encodes a member of the Rho family of GTPases. The encoded protein is localized to the outer mitochondrial membrane and plays a role in mitochondrial trafficking and fusion-fission dynamics. |  |
| *RHBDL1* | This gene encodes a protein similar to Rhomboid in Drosophila which is involved in signalling in the Spitz/epidermal growth factor receptor/mitogen-activated protein kinase pathway. The Rhomboid family of proteins consists of intramembrane serine proteases containing several transmembrane domains. |  |
| *STUB1* | This gene encodes a protein containing tetratricopeptide repeat and a U-box that functions as a ubiquitin ligase/cochaperone. The encoded protein binds to and ubiquitinates shock cognate 71 kDa protein (Hspa8) and DNA polymerase beta (Polb), among other targets. | Spinocerebellar ataxia, autosomal recessive, 16 (SCAR16) [MIM:615768]: Spinocerebellar ataxia defines a clinically and genetically heterogeneous group of cerebellar disorders. Patients show progressive incoordination of gait and often poor coordination of hands, speech and eye movements, due to degeneration of the cerebellum with variable involvement of the brainstem and spinal cord. SCAR16 is characterized by truncal and limb ataxia resulting in gait instability. Additionally, patients may show dysarthria, nystagmus, spasticity of the lower limbs, and mild peripheral sensory neuropathy. |
| *FBXL16* | Members of the F-box protein family, such as FBXL16, are characterized by an approximately 40-amino acid F-box motif. SCF complexes, formed by SKP1 (MIM 601434), cullin (see CUL1; MIM 603134), and F-box proteins, act as protein-ubiquitin ligases. F-box proteins interact with SKP1 through the F box, and they interact with ubiquitination targets through other protein interaction domains (Jin et al., 2004 [PubMed 15520277]).[supplied by OMIM, Mar 2008] |  |
| *METRN* | Meteorin regulates glial cell differentiation and promotes the formation of axonal networks during neurogenesis (Nishino et al., 2004 [PubMed 15085178]). |  |
| *NARFL* | **-** |  |
| *MSLN* | This gene encodes a preproprotein that is proteolytically processed to generate two protein products, megakaryocyte potentiating factor and mesothelin. Megakaryocyte potentiating factor functions as a cytokine that can stimulate colony formation of bone marrow megakaryocytes. Mesothelin is a glycosylphosphatidylinositol-anchored cell-surface protein that may function as a cell adhesion protein. |  |
| *CHTF18* | This gene encodes a protein which is a component of a replication factor C (RFC) complex, which loads proliferating cell nuclear antigen (PCNA) on to DNA during the S phase of cell cycle. The encoded protein may interact with other proteins, including RFC complex 3, to form a clamp loader complex that plays a role in sister chromatid cohesion during metaphase-anaphase transition. |  |
| *GNG13* | Heterotrimeric G proteins, which consist of alpha (see MIM 139320), beta (see MIM 139380), and gamma subunits, function as signal transducers for the 7-transmembrane-helix G protein-coupled receptors. GNG13 is a gamma subunit that is expressed in taste, retinal, and neuronal tissues and plays a key role in taste transduction (Li et al., 2006 [PubMed 16473877]). |  |
| *LMF1* | The protein encoded by this gene resides in the endoplasmic reticulum, and is involved in the maturation and transport of lipoprotein lipase through the secretory pathway. Mutations in this gene are associated with combined lipase deficiency. | Combined lipase deficiency (CLD) [MIM:246650]: Characterized by repeated episodes of pancreatitis, tuberous xanthomas and lipodystrophy and is caused by deficiency of both lipoprotein lipase (LPL) and hepatic triglyceride lipase (HTGL). {ECO:0000269 PubMed:17994020}. |

**Table S1.** Genes located within the a two copy DNA gain in the 16p13.3 region of patient 2

|  | Condition 1 | Condition 2 | reporter | t | df | standard error of difference | two-tailed P value | statistical  significance |
| --- | --- | --- | --- | --- | --- | --- | --- | --- |
|  | SOX8_WT | SOX8_E156D | *NR5A1-*luc | 3.0092 | 4 | 10.900 | 0.0396 | statistically significant |
|  | SOX8_WT | SOX8_E156D | *DMRT1-*luc | 6.0123 | 4 | 2.467 | 0.0039 | very statistically significant |
|  | SOX8_WT | SOX8_E156D | *AMH-*luc | 0.6344 | 4 | 41.958 | 0.5603 | not statistically significant |
|  | NR5A1_WT | NR5A1_WT +SOX8_WT | *AMH-*luc | 1.2647 | 4 | 49.394 | 0.2746 | Not statistically significant |
|  | NR5A1_WT | NR5A1_WT +SOX8_E156D | *AMH-*luc | 1.5572 | 4 | 50.071 | 0.1944 | not statistically significant |
|  | SOX8_WT | SOX8_E156D | *Tesco-*luc | 0.0222 | 8 | 5.238 | 0.9829 | not statistically significant |
|  | NR5A1_WT | NR5A1_WT +SOX8_WT | *Tesco-*luc | 4.8684 | 8 | 7.674 | 0.0012 | very statistically significant |
|  | NR5A1_WT | NR5A1_WT +SOX8_E156D | *Tesco-*luc | 0.032 | 8 | 2.503 | 0.566 | not statistically significant |
|  | NR5A1_WT +SOX8_WT | NR5A1_WT +SOX8_E156D | *Tesco-*luc | 7.6219 | 8 | 8.072 | < 0.0001 | extremely statistically significant |
|  | SOX8_WT | SOX8_D382N | *DMRT1-*luc | 2.4381 | 4 | 42.433 | 0.0714 | not quite statistically significant |
|  | SOX8_WT | SOX8_G359R | *DMRT1-*luc | 22.2073 | 4 | 3.858 | < 0.0001 | extremely statistically significant |
|  | SOX8_WT | SOX8_G378S | *DMRT1-*luc | 130.95 | 4 | 0.522 | < 0.0001 | extremely statistically significant |
|  | SOX8_WT | SOX8_A373S | *DMRT1-*luc | 9.5728 | 4 | 2.649 | 0.0007 | extremely statistically significant |
|  | SOX8_WT | SOX8_K241T | *DMRT1-*luc | 2.3273 | 4 | 28.563 | 0.0805 | not quite statistically significant |
|  | SOX8_WT | SOX8_R8_S9del | *DMRT1-*luc | 10.3443 | 4 | 7.318 | 0.0005 | extremely statistically significant |
|  | SOX8_WT | SOX8_A32V | *DMRT1-*luc | 2.0373 | 4 | 34.111 | 0.1113 | not statistically significant |
|  | SOX8_WT | SOX8_P242L | *DMRT1-*luc | 3.1813 | 4 | 19.211 | 0.0335 | statistically significant |
|  | SOX8_WT | SOX8_A416T | *DMRT1-*luc | 18.9369 | 4 | 3.743 | <0.0001 | extremely statistically significant |
|  | SOX8_WT | SOX8_S267L | *DMRT1-*luc | 43.3081 | 4 | 2.159 | <0.0001 | extremely statistically significant |
|  | SOX8_WT | SOX8_V282I | *DMRT1-*luc | 8.1397 | 4 | 8.032 | 0.0012 | very statistically significant |
|  | SOX8_WT | SOX8_D382N | *AMH-*luc | 33.3372 | 4 | 2.540 | <0.0001 | extremely statistically significant |
|  | SOX8_WT | SOX8_G359R | *AMH-*luc | 20.0014 | 4 | 4.216 | <0.0001 | extremely statistically significant |
|  | SOX8_WT | SOX8_G378S | *AMH-*luc | 18.9982 | 4 | 3.171 | <0.0001 | extremely statistically significant |
|  | SOX8_WT | SOX8_A373S | *AMH-*luc | 3.3776 | 4 | 14.063 | 0.0278 | statistically significant |
|  | SOX8_WT | SOX8_K241T | *AMH-*luc | 11.1779 | 4 | 3.268 | 0.0004 | extremely statistically significant |
|  | SOX8_WT | SOX8_R8_S9del | *AMH-*luc | 4.0183 | 4 | 10.305 | 0.0159 | statistically significant |
|  | SOX8_WT | SOX8_A32V | *AMH-*luc | 3.2961 | 4 | 6.790 | 0.0300 | statistically significant |
|  | SOX8_WT | SOX8_P242L | *AMH-*luc | 5.6179 | 4 | 5.807 | 0.0049 | very statistically significant |
|  | SOX8_WT | SOX8_A416T | *AMH-*luc | 63.2407 | 4 | 1.421 | <0.0001 | extremely statistically significant |
|  | SOX8_WT | SOX8_S267L | *AMH-*luc | 8.8003 | 4 | 4.872 | 0.0009 | extremely statistically significant |
|  | SOX8_WT | SOX8_V282I | *AMH-*luc | 9.8394 | 4 | 5.080 | 0.0006 | extremely statistically significant |
|  | SOX8_WT | SOX8_D382N | *NR5A1-*luc | 0.3468 | 4 | 6.291 | 0.7462 | not statistically significant |
|  | SOX8_WT | SOX8_G359R | *NR5A1-*luc | 79.9119 | 4 | 1.114 | <0.0001 | extremely statistically significant |
|  | SOX8_WT | SOX8_G378S | *NR5A1-*luc | 2.7887 | 4 | 13.191 | 0.0494 | statistically significant |
|  | SOX8_WT | SOX8_A373S | *NR5A1-*luc | 2.7854 | 4 | 13.592 | 0.0495 | statistically significant |
|  | SOX8_WT | SOX8_K241T | *NR5A1-*luc | 1.6967 | 4 | 32.205 | 0.1650 | not statistically significant |
|  | SOX8_WT | SOX8_R8_S9del | *NR5A1-*luc | 76.5514 | 4 | 1.085 | <0.0001 | extremely statistically significant |
|  | SOX8_WT | SOX8_A32V | *NR5A1-*luc | 11.1284 | 4 | 4.208 | 0.0004 | extremely statistically significant |
|  | SOX8_WT | SOX8_P242L | *NR5A1-*luc | 1.4447 | 4 | 8.713 | 0.2220 | not statistically significant |
|  | SOX8_WT | SOX8_A416T | *NR5A1-*luc | 29.5317 | 4 | 2.550 | <0.0001 | extremely statistically significant |
|  | SOX8_WT | SOX8_S267L | *NR5A1-*luc | 5.4479 | 4 | 7.085 | 0.0055 | very statistically significant |
|  | SOX8_WT | SOX8_V282I | *NR5A1-*luc | 4.7702 | 4 | 15.293 | 0.0088 | very statistically significant |

**Table S2**.
